# Knoevenagel C=C Metathesis Enabled Glassy Vitrimers with High Rigidity, Toughness, and Malleability

**DOI:** 10.1021/jacs.4c03503

**Published:** 2024-05-28

**Authors:** Sheng Wang, Hongzhi Feng, Bofan Li, Jason Y. C. Lim, Wendy Rusli, Jin Zhu, Nikos Hadjichristidis, Zibiao Li

**Affiliations:** † Institute of Sustainability for Chemicals, Energy and Environment (ISCE2), Agency for Science, Technology and Research (A*STAR), 1 Pesek Road, Jurong Island, Singapore 627833, Republic of Singapore; ‡ Key Laboratory of Bio-Based Polymeric Materials Technology and Application of Zhejiang Province, Laboratory of Polymers and Composites, Ningbo Institute of Materials Technology and Engineering, Chinese Academy of Sciences, Ningbo 315201, P. R. China; § Institute of Materials Research and Engineering (IMRE), Agency for Science, Technology and Research (A*STAR), 2 Fusionopolis Way, Innovis #08-03, Singapore 138634, Republic of Singapore; ∥ Polymer Synthesis Laboratory, Physical Sciences and Engineering Division, KAUST Catalysis Center, King Abdullah University of Science and Technology (KAUST), Thuwal 23955, Saudi Arabia; ⊥ Department of Materials Science and Engineering, National University of Singapore, Singapore 117576, Republic of Singapore

## Abstract

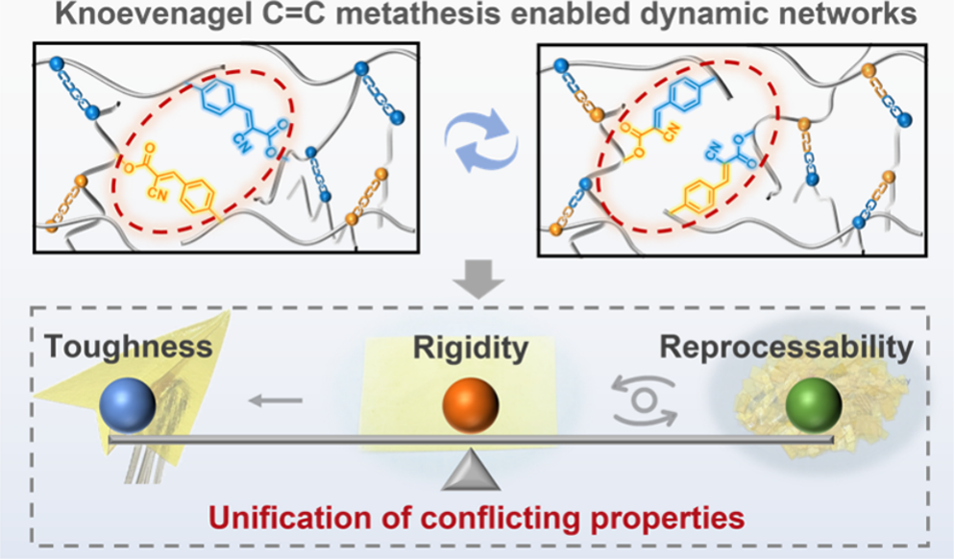

Thermosets, characterized by their permanent cross-linked networks, present significant challenges in recyclability and brittleness. In this work, we explore a polarized Knoevenagel C=C metathesis reaction for the development of rigid yet tough and malleable thermosets. Initial investigation on small molecule model reactions reveals the feasibility of conducting the base-catalyzed C=C metathesis reaction in a solvent-free environment. Subsequently, thermosetting poly(α-cyanocinnamate)s (PCCs) were synthesized via Knoevenagel condensation between a triarm cyanoacetate star and a dialdehyde. The thermal and mechanical properties of the developed PCCs can be easily modulated by altering the structure of the dialdehyde. Remarkably, the introduction of ether groups into the PCC leads to a combination of high rigidity and toughness with Young’s modulus of ∼1590 MPa, an elongation at break of ∼79%, and a toughness reaching ∼30 MJ m^3^. These values are competitive to traditional thermosets, in Young’s modulus but far exceed them in ductility and toughness. Moreover, the C=C metathesis facilitates stress relaxation within the bulk polymer networks, thus rendering PCCs excellent malleability and reprocessability. This work overcomes the traditional limitations of thermosets, introducing groundbreaking insights for the design of rigid yet tough and malleable thermosets, and contributing significantly to the sustainability of materials.

## Introduction

Thermosets play an essential and irreplaceable role in various high-demand applications, including antifouling and anticorrosive coatings, windmills, automotive engineering, and aerospace.^1−3^ They are rigid, dimensionally, thermally, and chemically stable materials due to their three-dimensional cross-linked networks. However, the *Achilles’ heel* of thermosets lies in their permanent cross-linked networks: they are extremely difficult to be reprocessed, recycled, and reshaped once fully cured, which is in stark contrast to end-of-use thermoplastics.^4^ The primary disposal methods for thermosets, predominantly incineration and landfill, exacerbate environmental pollution and constitute gross mismanagement of resources.

Incorporating dynamic covalent bonds into cross-linked polymer networks to produce covalent adaptable networks (CANs) represents an effective method to provide recyclability and/or reprocessability to thermosets.^5−8^ CANs possess the ability to rearrange their network topology through dynamic covalent bond exchange triggered by external stimuli. CANs with associative exchange mechanisms, also known as vitrimers,^9^ have attracted significant attention due to their persistent cross-link density, which provides resistance against solvent exposure and thermal dissociation.^10,11^ Various associative dynamic covalent reactions, including transesterification,^9^ nucleophilic aromatic substitution,^12^ diketoenamine exchange,^13^ imine exchange,^14^ silyl ether exchange,^15^ vinylogous urethane exchange,^16^ dioxaborolane metathesis,^17^ acetal exchange,^18^ and others,^10^ have been incorporated into polymer networks to produce these materials. However, on the one hand, most chemical entities in these exchange reactions require free hydroxyls or amines, which will inevitably cause side reactions under external stimuli to yield unwanted permanent cross-links.^17,19^ On the other hand, like traditional thermosets, glassy vitrimers often exhibit brittleness due to their three-dimensional cross-linked networks.^20^ Toughness is a critical characteristic for engineering plastics or structural materials, as it helps prevent stress cracking during practical applications.

The Knoevenagel reaction is a well-established method for forming C=C bonds with electron-withdrawing groups (EWGs).^21,22^ This reaction involves the condensation of aldehydes or ketones with active methylene compounds. A notable feature of the resulting Knoevenagel adducts is the enhanced conjugation imparted by the EWGs, which establishes a push–pull electronic system while simultaneously amplifying the double bond’s polarity.^23^ Such well-conjugated systems is suitable for preparing glassy polymers with high rigidity. In addition, the highly polarized C=C bond is capable of reversible formation,^24,25^ or involvement in exchange reactions with active methylene compounds^26,27^ or thiols,^28^ or metathesis reactions with imine bonds.^23,29^ However, the direct metathesis reaction involving the Knoevenagel C=C bond itself has not been previously reported.

**Figure 1:**
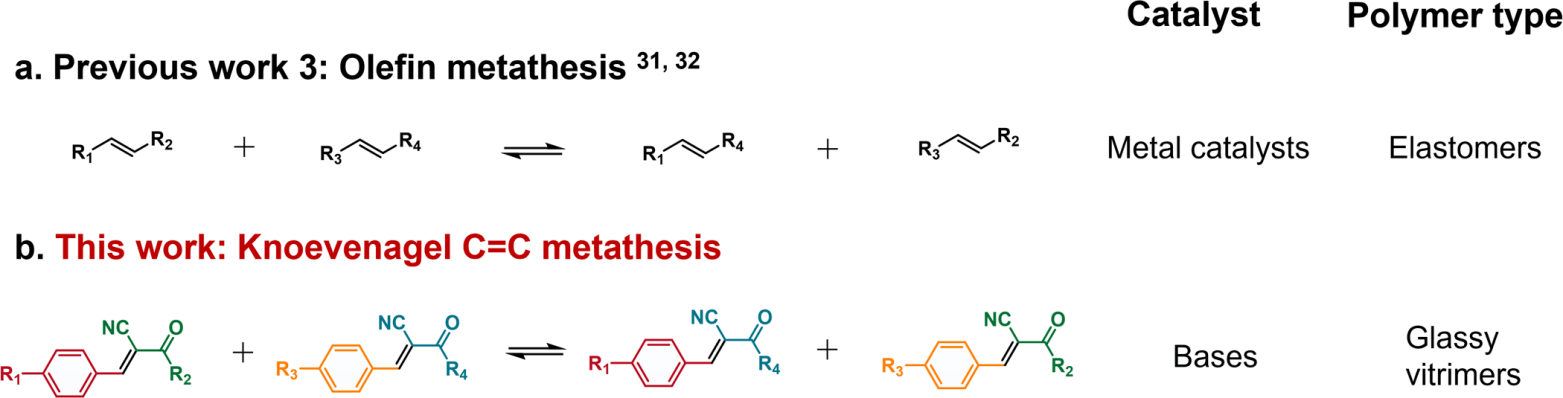
Schematic representation of the comparison of (a) previous works olefin metathesis, and (b) this work Knoevenagel C=C metathesis.

Here, we explore the Knoevenagel C=C metathesis for constructing vitrimers to address the trade-off between material performance and reprocessability of dynamic cross-linked polymers. Glassy vitrimer poly(α-cyanocinnamate)s (PCCs) with conjugated and dynamic Knoevenagel C=C bonds were successfully fabricated under basic catalysis and demonstrated high rigidity with Young’s modulus ranging from 1530 to 2280 MPa and toughness ranging from 9.58 to 30.3 MJ m^–3^; this is in contrast to conventional olefin metathesis,^30^ which often involves a metal catalyst like Grubbs’ Ru catalyst to produce elastomeric vitrimers^31,32^ (Figure 1). Model experiments utilizing small molecules demonstrated the feasibility of C=C metathesis reactions catalyzed by bases. The polymer networks were then synthesized via the facile Knoevenagel reaction between a triarm cyanoacetate star and a dialdehyde, with the added benefit of tuning the polymer properties through structural modulation of dialdehydes. Furthermore, stress relaxation experiments revealed the Arrhenius flow characteristics and vitrimeric nature of the developed PCC networks.

## Results and Discussion

### Model Knoevenagel C=C Metathesis Reaction

**Figure 2:**
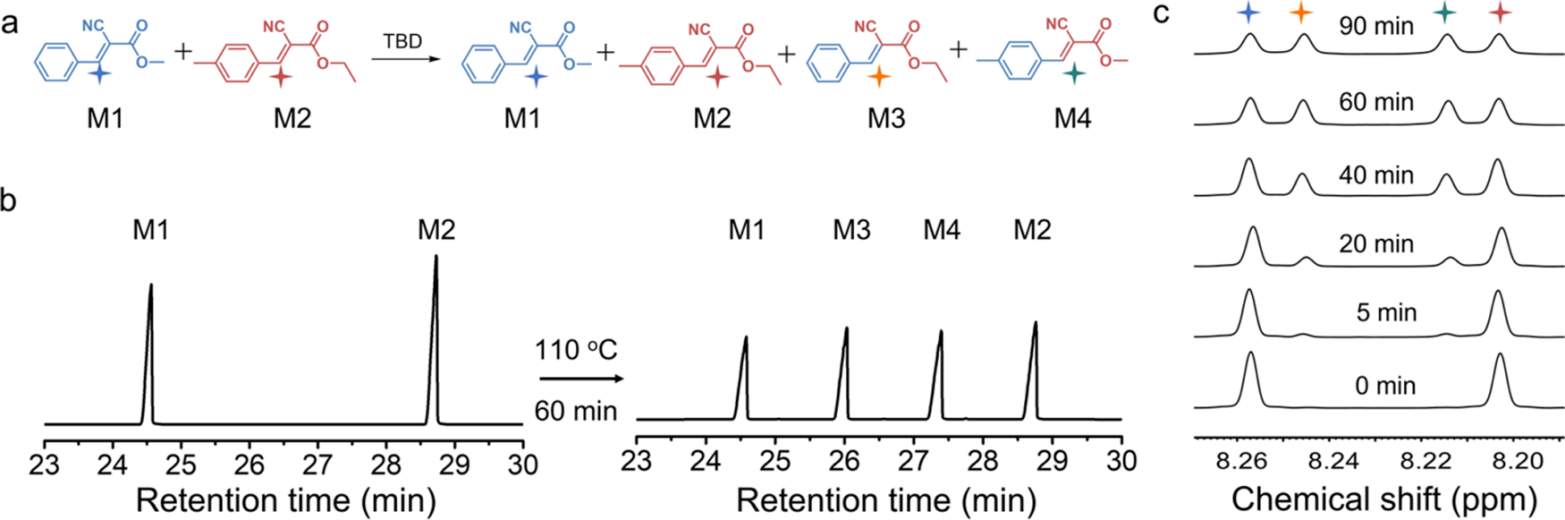
Knoevenagel C=C metathesis model reaction. (a) Schematic representation of the C=C metathesis reaction involving Knoevenagel adducts M1 and M2 catalyzed by TBD, leading to the formation of M1, M2, M3, and M4. (b) Gas chromatography traces illustrating the progression of the C=C metathesis reaction between an equivalent amount of M1 and M2 before and after 60 min at 110 °C, with retention times for M1, M2, M3, and M4 recorded at 24.6, 28.8, 26.0, and 27.3 min, respectively. 1 mol % TBD was used as a catalyst (c) ^1^H NMR (500 MHz, CDCl_3_, 22 °C) spectra of the C=C metathesis reaction between an equivalent amount of M1 and M2 with a 1 mol % TBD catalyst, showing changes over various durations at 110 °C.

Small molecule model reactions were designed to study the Knoevenagel C=C metathesis reaction (Figure 2a). Two Knoevenagel adducts, M1 and M2, were synthesized via the Knoevenagel condensation of benzaldehyde with methyl cyanoacetate and p-tolualdehyde with ethyl cyanoacetate, respectively (Figure S1). These two adducts underwent purification through sequential washing and recrystallization. Their purity was confirmed by mass spectrometry, gas chromatography, and ^1^H and ^13^C nuclear magnetic resonance (NMR) spectroscopy, indicating no detectable residual monomers or other impurities (Figures S2–S5). Strict moisture control was applied to prevent hydrolysis during exchange reactions: initial monomers, solvents, and reaction vials were thoroughly dehydrated, repeatedly evacuated, and filled with argon. Surprisingly, upon heating a mixture of an equivalent amount of M1 and M2 with 1 mol % TBD (catalyst) at 110 °C for 10 min without a solvent, Knoevenagel adducts M3 and M4 were detected (Figures S6–S10). After 60 min, the mixture contained a similar molar amount of all four Knoevenagel adducts without any evidence of dissociated products derived from the Knoevenagel adducts (Figures 2b and S10), indicating the occurrence of a metathesis reaction.

Further evidence of the C=C metathesis was obtained from ^1^H NMR spectra recorded at 110 °C for various durations (Figures 2c and S11). Before the reaction, ^1^H NMR showed proton signals in −CH=C(CN)- at. for M1 and 8.203 ppm for M2, −O–CH_3_ of 3.932 ppm for M1 (Figures 2c and S11). Upon heating the mixtures, the appearance of new proton signals at about 8.245 and 8.214 ppm can be attributed to the protons in −CH=C(CN)- of M3 and M4, respectively, and 3.918 ppm to the proton in −O–CH_3_ of M4. The relative intensities of these proton signals were increased with reaction time. In contrast, the intensities of the peaks corresponding to the initial monomers, M1 and M2, show a gradual decrease over time. An associative mechanism for the model C=C metathesis reaction is proposed (Figure S12), involving a nucleophilic attack by TBD on M1, generating a stabilized intermediate anion that can then nucleophilically attack M2, forming a four-membered ring intermediate. Subsequent ring opening occurs to yield the metathesis products.

To gain deeper insights into the developed C=C metathesis reaction, a kinetic study was conducted by reacting M1 with an equivalent amount of M2 in the presence of 1 mol % TBD in CDCl_3_. The real-time evolution of the exchange reaction was in situ monitored through ^1^H NMR spectroscopy at different temperatures. The progress of the reaction was tracked by measuring the decrease in intensity of proton signals in the −CH=C(CN)– of M1 in the ^1^H NMR spectra over time (Figure S13a). A linear relationship between (1/[M1]-1/[M1]_0_) and time was observed in the early stages (1500 s) of the reaction (Figure S13b), aligning with a second-order kinetics. Furthermore, an Arrhenius relationship between reaction kinetics and temperature was observed with an activation energy (*E*_a_) value of 34 kJ mol^–1^ (Figure S13c).

In addition, we investigated the effect of catalyst nucleophilicity on the C=C metathesis reaction. Triethylamine (TEA, p*K*_a_: 10.7) and *N*,*N*-diisopropylethylamine (DIPEA: 10.98) with similar p*K*_a_ but distinguished nucleophilicity were selected as the catalysts. Specifically, M1 was reacted with an equivalent amount of M2 in the presence of 0.2 mol % TEA or DIPEA in DMSO-*d*_6_, and the real-time evolution of the exchange reaction was monitored in situ through ^1^H NMR spectroscopy at 25 °C. The reaction kinetics for different catalysts in these model reactions were calculated (Figure S14). It was observed that DIPEA was significantly less effective than TEA, indicating the critical role of catalyst nucleophilicity in driving the C=C metathesis reaction.

### Poly(α-cyanocinnamate)s (PCCs) Synthesis

**Figure 3:**
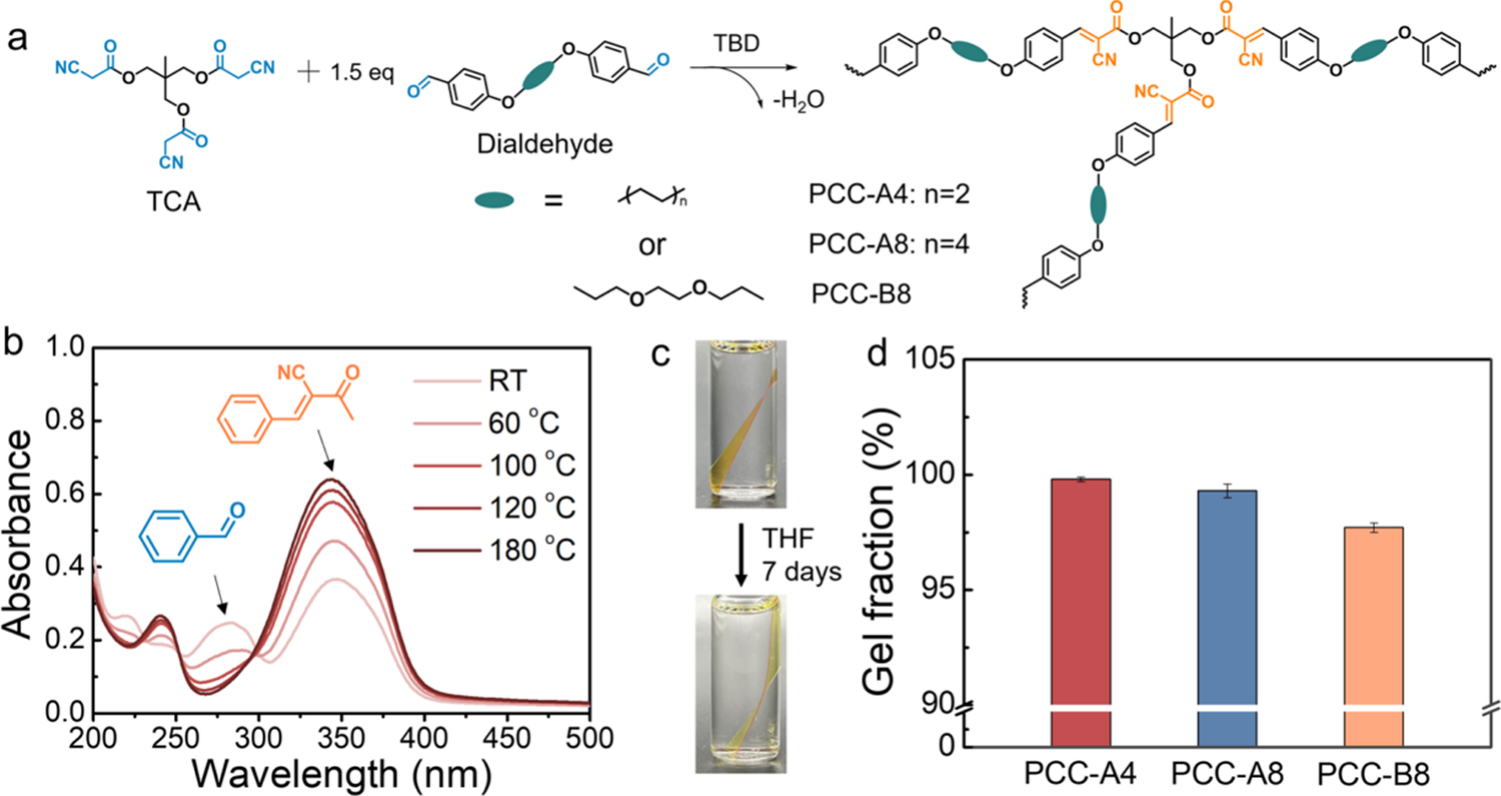
Polymer synthesis. (a) PCCs were synthesized from a triarm cyanoacetate (TCA) star and an alkyl diaromatic aldehyde (A4 or A8) or an ether-based diaromatic aldehyde (B8) with an equimolar ratio of active methylene and aldehyde groups through Knoevenagel polycondensation using triazabicyclodecene (TBD) as a catalyst at 5 mol % relative to C=C bonds. The resulting polymers are designated as PCC-A4, PCC-A8, and PCC-B8, respectively; (b) temperature-dependent UV–vis absorbance spectra of a mixture containing A4, TPA, and TBD on a quartz sheet; (c) comparative images of PCC-A4 (40 mg) before and after immersion in THF (4 mL) for 7 days; (d) gel fractions of PCCs extracted in THF.

After the successful observation of the C=C metathesis reaction in the preliminary model experiments, we applied it for the development of malleable thermosets. Figure 3a illustrates the synthesis of thermosetting PCCs via Knoevenagel polycondensation. This process involves the reaction of a triarm cyanoacetate (TCA) star with a dialdehyde in an equimolar ratio of active methylene and aldehyde groups, using TBD as a catalyst at 5 mol % relative to C=C bonds. Basically, three dialdehyde derivatives, designated as A4, A8, and B8, were synthesized via condensation reactions involving halogen and hydroxyl functionalities (Figures S15–S22). The alteration in the hydrocarbon chain length from A4 to A8 facilitated the modulation of cross-link density and structural flexibility in the resulting polymers. In compound B8, an ether linkage, reflecting the chain length of A8, was introduced to increase the segmental mobility within the PCC networks. Additionally, the TCA^26^ was synthesized through an esterification reaction between a cyanoacetic acid and a trimethylolethane (Figure S23). Subsequently, TCA, a dialdehyde, and a catalyst TBD were mixed in THF (Table S1), followed by slowly evaporating solvent at RT and precuring at 60 °C for 30 min to enhance the reaction degree and prevent side reactions of reactive groups that are likely at higher temperatures. The resulting precured polymer was then hot-pressed at 170 °C to further increase the cross-linking degree and expulsion of any generated water during the process. This curing method produced a defect-free and transparent PCC film (Figure S24).

Ultraviolet–visible (UV–vis) and Fourier-transform infrared (FTIR) spectroscopy were utilized to confirm the formation of C=C bonds throughout the curing process. The temperature-dependent UV–vis spectra, shown in Figure 3b, revealed a notable absorption peak at approximately 345 nm at room temperature, indicating the formation of conjugated Knoevenagel adducts. This absorption peak’s intensity increased with rising temperature. At the same time, the absorbance peak for benzaldehyde decreased progressively with increasing temperature, and finally disappeared. Figure S25a presents the FTIR spectra of starting monomers A4 and TCA and the polymer network PCC-A4. After curing, the disappearance of the C=O stretching vibration at 1677 cm^–1^, characteristic of the aldehyde group in A4, was observed. Additionally, a redshift in the cyano group’s stretching vibration (from 2265 to 2220 cm^–1^) caused by the conjugated adduct formation was detected. Similar patterns were evident in the FTIR spectra of both PCC-A8 and PCC-B8 (Figure S25), indicative of the successful Knoevenagel polycondensation in the curing process. Moreover, the synthesized PCCs exhibited insolubility in tetrahydrofuran (THF), as shown in Figures 3c and S26. The gel fractions exceed 96% for all three samples after extraction in different solvents (Figure 3d and Table S2). These findings highlight the successful formation of Knoevenagel C=C bonds and cross-linked networks during the curing process.

### Thermal and Mechanical Properties

**Figure 4:**
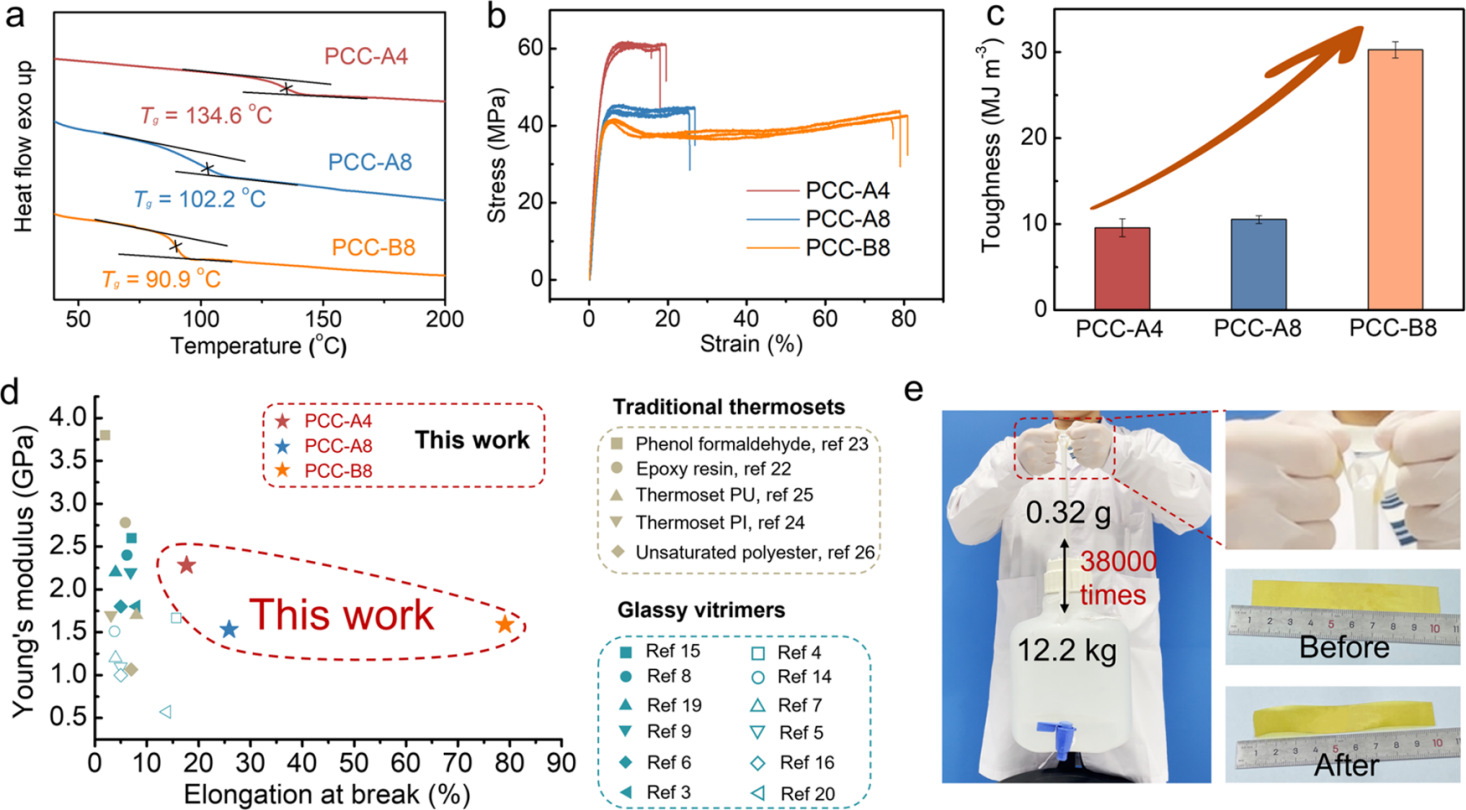
Thermal and mechanical properties of PCCs. (a) DSC thermographs, (b) stress–strain diagrams, and (c) toughness values of the developed PCCs; (d) An Ashby plot comparing Young’s modulus and elongation at break for PCCs against traditional thermosets and previously reported glassy vitrimers (*T*_g_ > 30 °C). Additional vitrimer types, traditional thermosets, detailed mechanical property data, and references are provided in Table S2; (e) digital photograph demonstrating that the PCC-B8 (0.32 g) can lift a 12.2 kg bucket. The appearance and dimension of samples before and after loading are recorded.

The thermal properties of the PCCs were carefully evaluated using differential scanning calorimetry (DSC), dynamic mechanical analysis (DMA), and thermogravimetric analysis (TGA). These polymers exhibited adjustable glass transition temperature (*T*_g_) values ranging from 90.9 °C for PCC-B8, 102.2 °C for PCC-A8, to 134.6 °C for PCC-A4, as determined from DSC thermograms (Figure 4a). PCC-A4 exhibited a higher *T*_g_ relative to PCC-A8 and PCC-B8, consistent with its higher cross-linking density and proportion of conjugated Knoevenagel adducts. In contrast, PCC-B8, with its flexible ether linkages, displayed the lowest *T*_g_, indicative of increased segmental mobility. This *T*_g_ trend was further confirmed by the tan delta curves from DMA temperature sweep tests (Figure S27). Besides, the molecular weight between cross-links (*M*_*c*_) was calculated from the rubbery plateau of storage modulus curves (Figure S27) to evaluate the cross-link density of PCCs. The calculated *M*_*c*_ was 2116, 2559, 2623 g mol^–1^ for PCC-A4, PCC-A8, and PCC-B8, respectively. PCC-A4 showed the highest cross-link density, which aligned with our design expectations, while PCC-A8 and PCC-B8 displayed comparable cross-link densities, validating the *T*_g_ values, which reflected variations in chain mobility. In addition, TGA results revealed that PCCs possess remarkable thermal stability with their initial degradation temperatures (*T*_d5%_) exceeding 358 °C (Figure S28).

To evaluate the mechanical properties, the PCC films were cut into rectangular strips and subjected to uniaxial tensile testing. PCCs displayed outstanding mechanical characteristics, combining high rigidity with toughness (Figures 4b,c and S29). At a strain rate of 5% min^–1^, PCC-A4 exhibited a Young’s modulus of 2280 MPa, an ultimate tensile strength of 62 MPa, an elongation at break of 17.7%, and a toughness of 9.58 MJ m^–3^. The mechanical properties of PCCs were found to be highly tunable: PCC-A8, with four additional methylene groups in each hydrocarbon chain compared to PCC-A4, became more ductile with a reduced Yound’s modulus of 1520 MPa and a higher elongation at break of 25.9%. Besides, the incorporation of an ether group in PCC-B8 significantly enhanced its elongation at break and toughness to 79.1% and 30.3 MJ m^–3^, respectively, with Young’s modulus and ultimate tensile strength comparable to PCC-A8. The yield behavior in stress–strain curves (Figure 4b) and ductile fracture surface observed in SEM images of tensile cross-section (Figure S30) further confirmed the PCC-B8′s ductility. Furthermore, the Young’s modulus and elongation at breaks of the developed PCCs were compared to those of glassy vitrimeric materials (*T*_g_ > 30 °C) with various dynamic bonds and traditional thermosets. As depicted in Figure 4d and Table S3, PCCs showed Young’s modulus comparable to these mechanically robust polymers, with substantially higher elongation at breaks.

The exceptional combination of high rigidity and toughness in PCC-B8 was demonstrated when a thin strip (approximately 0.32 g, 0.15 mm thick, 1.5 cm wide, and 10.2 cm long) successfully lifted a 12.2 kg bucket, as shown in Figure 4e and Video S1. This demonstrates a load-bearing capacity of roughly 38,000 times the PCC-B8 strip’s weight. The strip showed no significant deformation except in the regions under stress.

### Dynamics within Polymer Networks

**Figure 5:**
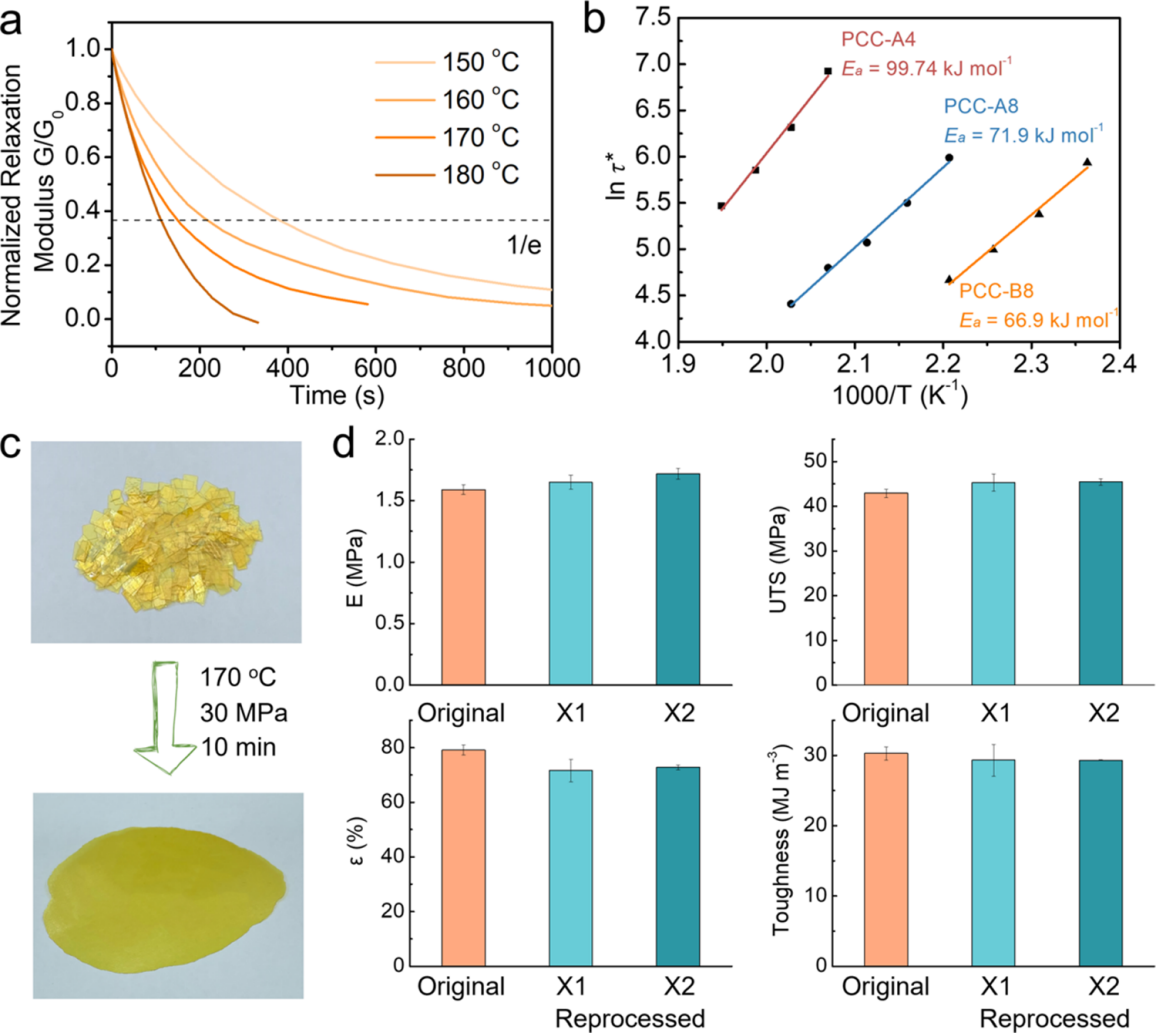
Malleability and reprocessabillity. (a) Normalized stress relaxation curves of PCC-B8 at temperatures ranging from 150 to 180 °C; (b) Arrhenius plots of PCC-A4, PCC-A8, and PCC-B8. (c) Images of forming reprocessed PCC-B8 film from chopped PCC-B8 pieces. (d) Tensile properties of original and two cycles reprocessed PCC-B8. Error bars correspond to standard deviation.

Despite their high cross-link density and robust mechanical strength, PCCs were found to be highly malleable, which is attributed to the C=C metathesis in the presence of the TBD catalyst. Stress relaxation analysis at elevated temperatures was performed to investigate the bond exchange in bulk and flow characteristics within polymer networks (Figures 5a and S31). All PCC samples present stress relaxation behavior at selected temperature ranges. Moreover, the results highlighted that the flexible chain length and chain segment mobility significantly influenced the stress relaxation rate. For example, at 210 °C, PCC-A8 showed a relaxation time of 210 s, approximately five times shorter than that of PCC-A4, which was 1016 s. Similarly, at 180 °C, PCC-A8 had a relaxation time of 399 s, while that of PCC-B8 was significantly shorter, at 106.2 s, about four times less. These values indicate that longer flexible chains and enhanced segment mobility facilitate stress relaxation in the polymer networks. Additionally, the effects of catalyst concentration and type on the stress relaxation behavior of the PCC networks were studied. When the loading of TBD in PCC-B8 was reduced from 5 to 3 mol%, or the catalyst was changed to 4-(dimethylamino)pyridine (DMAP), which possesses relatively weaker basicity and nucleophilicity, the relaxation rate of the PCC-B8 was observed to decrease (Figure S32a,b). This adjustment helps in harnessing the catalyst to modulate the rheological properties of the PCC networks. Conversely, when TEA was used as the catalyst, the polymer network did not exhibit stress relaxation behavior (Figure S32c). This likely results from the volatilization of TEA during the curing process, further underscoring the critical role of the catalyst in the C=C metathesis reaction.

The activation energy (*E*_a_) values for the C=C metathesis in the PCC networks were calculated using the Arrhenius equation (eq S7),^9^ considering the relaxation times at various temperatures (Figure 5b). The *E*_a_ values for PCC-A4, PCC-A8, and PCC-B8 were determined to be 99.7, 71.9, and 66.9 kJ mol^–1^, respectively. These values are comparable to those for associative exchange reactions in previously reported vitrimer systems (Table S3). Additionally, the topology freezing transition temperature (*T*_v_), where the viscosity is expected to reach 10^6^ MPa·s, was theoretically extrapolated from the Arrhenius plot.^33^ The estimated *T*_v_ values were 117 °C for PCC-A4, 56 °C for PCC-A8, and 33 °C for PCC-B8, which are above room temperature yet below the respective *T*_g_ of the PCCs (Table S3). Besides, the *T*_v_ values are consistent with the trends observed for the cross-link density and chain mobility of three PCCs.^34^

The rapid stress relaxation above the *T*_g_ provides PCCs with remarkable reprocessability, as demonstrated in PCC-B8. At 160 °C under 30 MPa pressure, PCC-B8 could be reprocessed from chopped samples to complete and transparent film (PCC-B8-R) in 10 min (Figure 5c). It is important to note that repeated chopping and hot-pressing molding might cause irreversible chain scission in the network, potentially altering the mechanical properties. However, PCC-B8 did not exhibit any significant degradation in its dynamic thermomechanical properties (Figure S33) and mechanical performance (Figures 5d and S34) after two reprocessing cycles. Interestingly, a slight increase in both *T*_g_, Young’s modulus, and a decrease in elongation at break were observed after reprocessing, possibly due to permanent cross-linking from side reactions^35^ such as oxidation of both cyano groups and double bonds, and cyclization of cyano groups under repeated reprocessing conditions. Furthermore, FTIR spectra of the reprocessed samples well aligned with those of the original samples (Figure S35), suggesting that the cross-linked network’s chemical structure remained unchanged after hot-pressing recycling.

## Conclusions

In this work, we report a base-catalyzed C=C metathesis reaction, which leads to the development of a class of vitrimers with a combination of high rigidity and toughness. The developed PCC networks, utilizing cost-effective and easily adaptable monomers, exhibit high gel fractions, and outstanding. thermal and mechanical properties while demonstrating excellent reprocessability. These features make them suitable for structural and related applications, contributing significantly to the sustainability of materials. Furthermore, we believe that incorporating various EWGs, using different aldehydes and catalysts, can profoundly affect the dynamics of the C=C metathesis reaction within the polymer networks. Based on this premise, further research can lead to the development of an extensive array of adaptable polymeric materials with unprecedented characteristics.

## Supporting Information Available

Materials, characterization methods, synthesis and experimental procedures, characterization data including ^1^H and ^13^C NMR spectra, gas chromatography traces, UV–vis and IR spectra, TGA thermograms, DMA curves, SEM imaging, stress relaxation curves, stress–strain curves (PDF)
Exceptional combination of rigidity and toughness in PCC-B8 was demonstrated when a thin strip (approximately 0.32 g, 0.15 mm thick, 1.5 cm wide, and 10.2 cm long) successfully lifted a 12.2 kg bucket, as shown in Figure 4e and Video S1 (MP4)
