# Supplementary material for: Knoevenagel C=C Metathesis Enabled Glassy Vitrimers with High Rigidity, Toughness, and Malleability
Source: J Am Chem Soc. 2024 May 28;146(23):16112–8. doi: 10.1021/jacs.4c03503 (PMC11177252; doi:10.1021/jacs.4c03503)
Supplement: Supplementary file 1 — ja4c03503_si_001.pdf [file ja4c03503_si_001.pdf]

Knoevenagel C=C Metathesis Enabled Glassy Vitrimers with High Rigidity, Toughness and Malleability

Sheng Wang[1]*, Hongzhi Feng[1,2], Bofan Li[1], Jason Y.C. Lim[3], Wendy Rusli[1], Jin Zhu[2], Nikos Hadjichristidis[4]*, Zibiao Li[1,3,5]*

[1]Institute of Sustainability for Chemicals, Energy and Environment (ISCE[2]), Agency for Science, Technology and Research (A*STAR), 1 Pesek Road, Jurong Island, Singapore 627833, Republic of Singapore.

[2]Key Laboratory of Bio-based Polymeric Materials Technology and Application of Zhejiang Province, Laboratory of Polymers and Composites, Ningbo Institute of Materials Technology and Engineering, Chinese Academy of Sciences, Ningbo 315201, P. R. China.

[3]Institute of Materials Research and Engineering (IMRE), Agency for Science, Technology and Research (A*STAR), 2 Fusionopolis Way, Innovis #08-03, Singapore 138634, Republic of Singapore;

[4]Polymer Synthesis Laboratory, Physical Sciences and Engineering Division, KAUST Catalysis Center, King Abdullah University of Science and Technology (KAUST), Thuwal, 23955, Saudi Arabia.

[5]Department of Materials Science and Engineering, National University of Singapore, Singapore, 117576, Republic of Singapore.

*Corresponding author. Email: wang_sheng@isce2.a-star.edu.sg (S.W.); Nikolaos.Hadjichristidis@kaust.edu.sa (N. H); lizb@imre.a-star.edu.sg (Z.L.)

Table of Contents

Materials and methods.. 3

Chemicals and solvents.. 3

General characterization methods.. 3

Model reactions for the C=C Metathesis.. 5

Synthesis and characterization of model compounds 5

Procedures for model reactions in bulk.. 10

Procedures for model reactions in solvents and kinetic studies.................. 12

Polymer synthesis and characterization.. 16

Synthesis of dialdehydes 16

Synthesis of a tri-arm cyanoacetate (TCA) 21

Preparation of poly(α-cyanocinnamate)s (PCCs). 21

Structural characterization.. 22

Swelling ratio and gel fraction tests. 23

Differential scanning calorimetry (DSC) tests... 24

Dynamic mechanical analysis (DMA) tests. 25

Calculation of the molecular weight between crosslinks (M_c) 25

Thermogravimetric analysis (TGA) tests. 26

Tensile tests and FESEM imaging of tensile cross-section 26

Malleability and reprocessability of PCC... 28

Stress relaxation measurements and activation energy calculation 28

Determination of the topology freezing temperature (T_v) 29

Reprocessing process 30

Supporting Tables.. 32

Refrences 34

Materials and methods

Chemicals and solvents

Acetonitrile (99.9 %), benzaldehyde (99%), dichloromethane (99.8%), 4-hydroxybenzaldehyde (98%), L-proline (99%), p-tolualdehyde (97%), sodium bicarbonate (99.5%), sodium hydroxide (98%), sodium sulfate (99%), tetrahydrofuran (99.9%), toluene (99.8%), 1,5,7-triazabicyclo[4.4.0]dec-5-ene (98%), triethylamine (99.5%) were purchased from Sigma-Aldrich. 1,2-Bis(2-chloroethoxy)ethane (98%), cyanoacetic acid (98%), 1,4-dichlorobutane (98%), 1,8-dichlorooctane (99%), ethyl cyanoacetate (99%), methyl cyanoacetate (99%), N,N-diisopropylethylamine (99%), p-toluenesulfonic acid monohydrate (98%), trimethylolethane (98%) were purchased from TCI. Acetone (99.5%) and isopropanol (99.9%) were purchased from AIK MOH. Ethanol (99.8%) was purchased from VWR Chemical. Deuterated chloroform (99.8%) and deuterated dimethyl sulfoxide (99.9%) were purchased from Cambridge Isotope Laboratories.

General characterization methods

^{1}H nuclear magnetic resonance (NMR) and ^{13}C NMR spectroscopy were performed on a JEOL JNM-ECA500II FT NMR System with deuterated chloroform or deuterated dimethyl sulfoxide as the solvent. Residual deuterated reagent or tetramethylsilane is used as an internal reference. Gas chromatography-mass spectrometry (GC-MS) was acquired on an Agilent 7890A under a controlled nitrogen atmosphere, with a flow rate set at 1 mL min^{-1}. The temperature program began with an initial hold at 50 °C for 1 minute, followed by a gradual increase at a rate of 5 °C min^{-1} up to 300 °C, where it was held constant for 5 minutes. Electrospray ionization–mass spectrometry (ESI-MS) was carried out using an Agilent LC-QTOF 6545B. The ultraviolet–visible (UV-vis) spectroscopy was performed on a Shimadzu UV-1800 double-beam spectrophotometer. Fourier transform Infrared (FT-IR) spectra were obtained on a Bruker Vertex 80v. Solid state spectra were recorded in attenuated total reflectance (ATR) mode and converted to absorbance spectra. Differential scanning calorimetry (DSC) tests were performed using a TA Instruments PDSC Q100. Samples were loaded into aluminum pans and scanned

against an empty reference pan. The dynamic mechanical analysis (DMA) tests were conducted using a TA Q800. The rectangular samples with dimensions of approximately 20 mm (length) x 5 mm (width) x 0.15 mm (thickness) were used for temperature sweep and stress relaxation tests. Thermogravimetric analysis (TGA) was carried out using a TA Instruments TGA Q500. Samples (around 5 mg) were tested from 50 to 700 °C with a heating rate of 10 °C min^{-1} under a nitrogen atmosphere. The mechanical properties were measured from uniaxial tensile tests using Instron 5569. The rectangular samples with dimensions of approximately 30 mm (length) x 5 mm (width) x 0.15 mm (thickness) were tested at RT with a cross-head speed of 5% min^{-1} until fracture. The field emission scanning electron microscope (FESEM) was imaged by a JEOL JSM 6700F. Before imaging, the samples were coated with a thin layer of gold using a gold sputtering equipment (Jeol JFC-1200).

Model reactions for the C=C Metathesis

Synthesis and characterization of model compounds

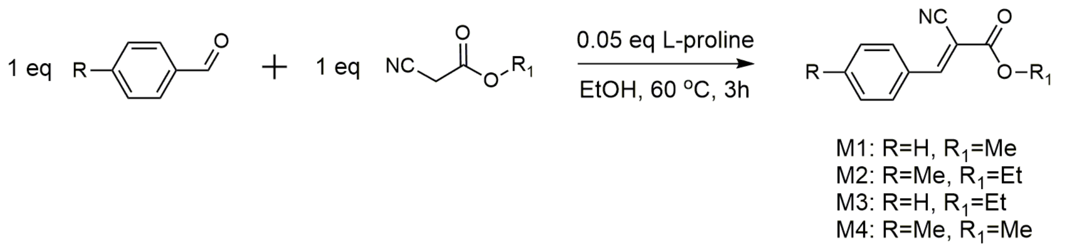

Figure S1 General synthetic route for the synthesis of model Knoevenagel compounds.

The synthesis of model Knoevenagel adducts was conducted employing the following general procedures. Cyanoacetate (1 eq), benzaldehyde (1 eq), and L-proline (0.05 eq) were dissolved in ethanol (1 g/ 3 mL) and subjected to a reaction at 60 °C for 3 hours. Upon completion of the reaction, the solution was cooled to 0 °C, facilitating the crystallization of the products. The crystalline precipitates were isolated through filtration and subsequently purified. This purification process involved repeated recrystallization steps, coupled with washes in cold ethanol, and additional filtration steps. The purified Knoevenagel compounds were then dried under a vacuum at 70 °C for a period of 24 hours to obtain the high-purity products.

Methyl (E)-2-cyano-3-phenylacrylate (M1): ^{1}H NMR (500 MHz, Chloroform-*d*) δ 8.23 (s, 1H), 7.96 (d, *J* = 4.9 Hz, 2H), 7.51 (dd, *J* = 31.0, 14.4 Hz, 3H), 3.91 (s, 3H). ^{13}C NMR (126 MHz, Chloroform-*d*) δ 162.80, 155.38, 133.28, 131.23, 130.96, 129.15, 115.31, 102.38, 53.26. MS (m/z): [M] calculated for $C_{11}H_9NO_2$, 187.0633; found, 187.0706.

Ethyl (E)-2-cyano-3-(p-tolyl)acrylate (M2): ^{1}H NMR (500 MHz, Chloroform-*d*) δ 8.20 (s, 1H), 7.88 (d, *J* = 8.6 Hz, 2H), 7.29 (s, 2H), 4.37 (t, *J* = 7.3 Hz, 2H), 2.42 (s, 3H), 1.50 – 1.29 (m, 3H). ^{13}C NMR (126 MHz, Chloroform-*d*) δ 162.67, 154.90, 144.57, 131.18, 129.95, 128.79, 115.70, 101.47, 62.50, 21.79, 14.10. MS (m/z): [M] calculated for $C_{13}H_{13}NO_2$, 215.0946; found, 215.0872.

Ethyl (E)-2-cyano-3-phenylacrylate (M3): ^{1}H NMR (500 MHz, Chloroform-*d*) δ 8.23 (s, 1H), 7.97 (s, 2H), 7.54 (d, *J* = 14.2 Hz, 1H), 7.49 (d, *J* = 11.2 Hz, 2H), 4.37 (dd, *J* = 13.6, 7.1 Hz, 2H), 1.39 (d, *J* = 12.8 Hz, 3H). ^{13}C NMR (126 MHz, Chloroform-*d*) δ 162.33, 154.90, 133.19, 131.35, 130.95, 129.16, 115.37, 102.89, 62.61, 14.04. MS (m/z): [M] calculated for $C_{12}H_{11}NO_2$, 201.0790; found, 201.0863.

Methyl (E)-2-cyano-3-(p-tolyl)acrylate (M4): ^{1}H NMR (500 MHz, Chloroform-*d*) δ 8.20 (s, 1H), 7.88 (s, 2H), 7.29 (s, 2H), 3.91 (s, 3H), 2.42 (s, 3H). ^{13}C NMR (126 MHz, Chloroform-*d*) δ 163.15, 155.14, 144.70, 131.20, 129.95, 128.69, 115.65, 100.96, 53.20, 21.77. MS (m/z): [M] calculated for $C_{12}H_{11}NO_2$, 201.0790; found, 201.0716.

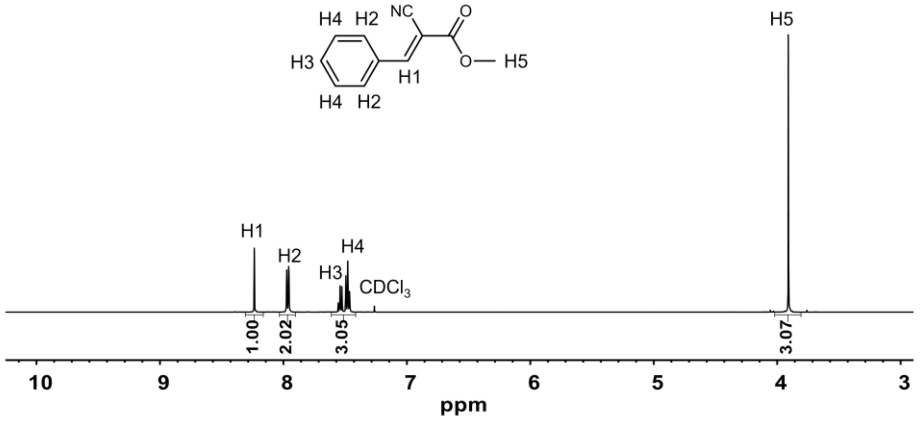

Figure S2 ^{1}H NMR (500 MHz, $CDCl_3$, 22 °C) spectrum of **M1**.

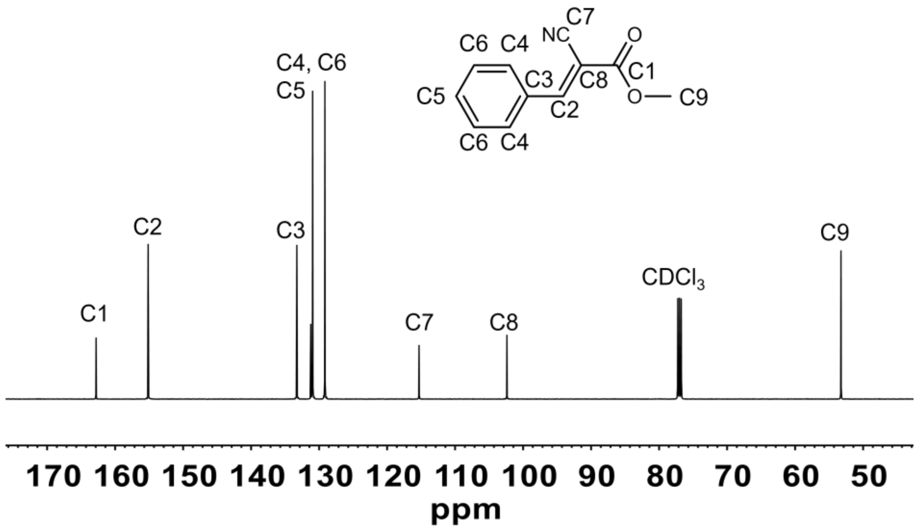

Figure S3 ^{13}C NMR (126 MHz, $CDCl_3$, 22 °C) spectrum of **M1**.

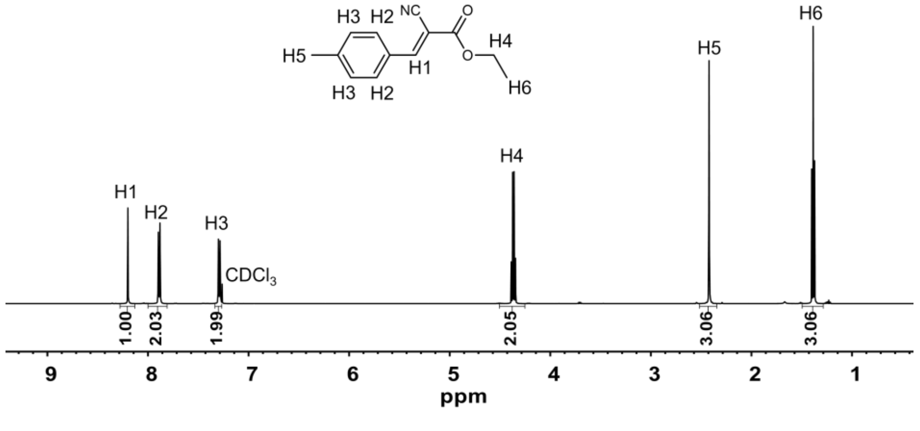

Figure S4 ^{1}H NMR (500 MHz, $CDCl_3$, 22 °C) spectrum of **M2**.

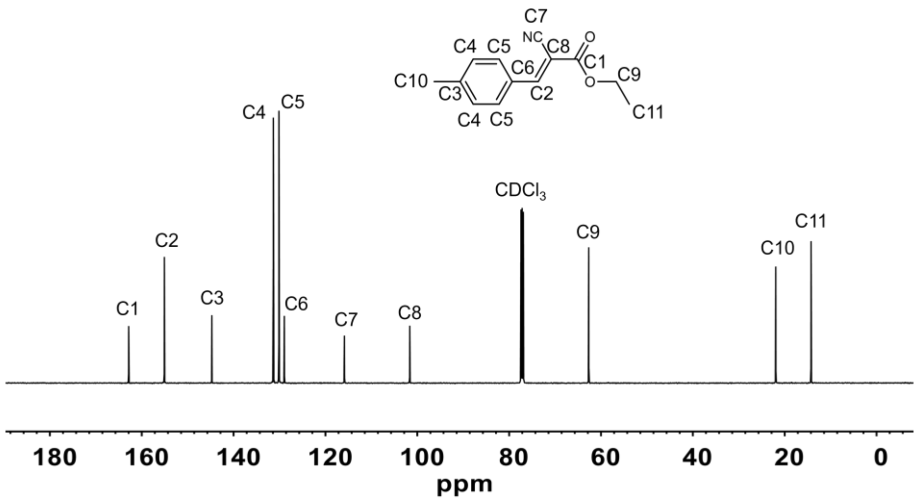

Figure S5 ^{13}C NMR (126 MHz, $CDCl_3$, 22 °C) spectrum of **M2**.

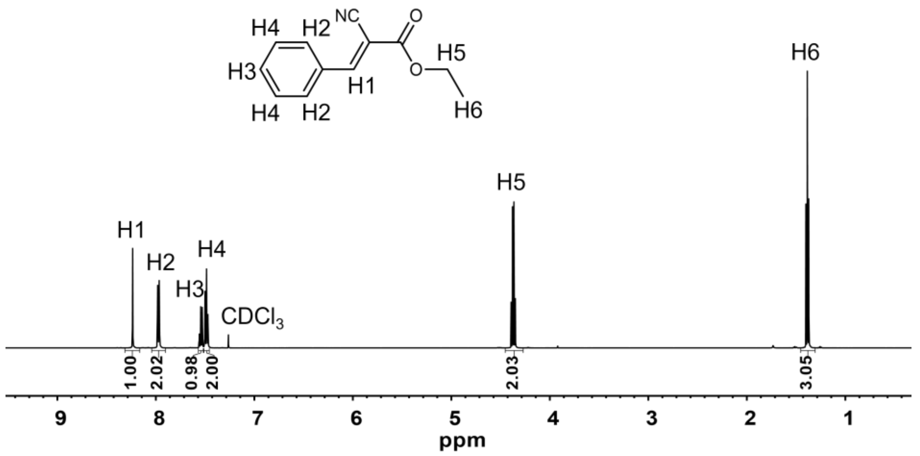

Figure S6 ^{1}H NMR (500 MHz, $CDCl_3$, 22 °C) spectrum of **M3**.

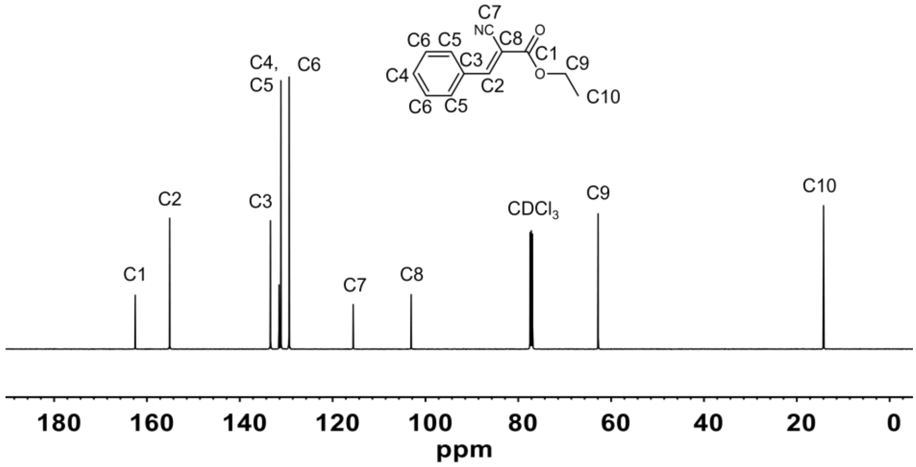

Figure S7 ^{13}C NMR (126 MHz, $CDCl_3$, 22 °C) spectrum of **M3**.

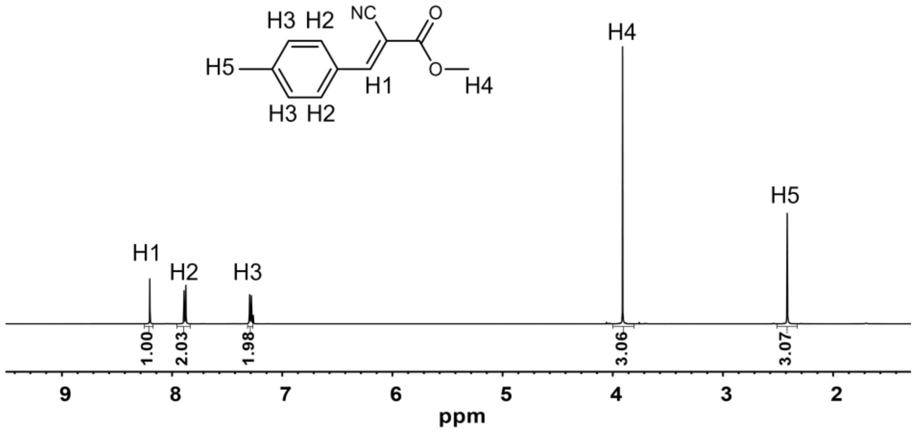

Figure S8 ^{1}H NMR (500 MHz, $CDCl_3$, 22 °C) spectrum of **M4**.

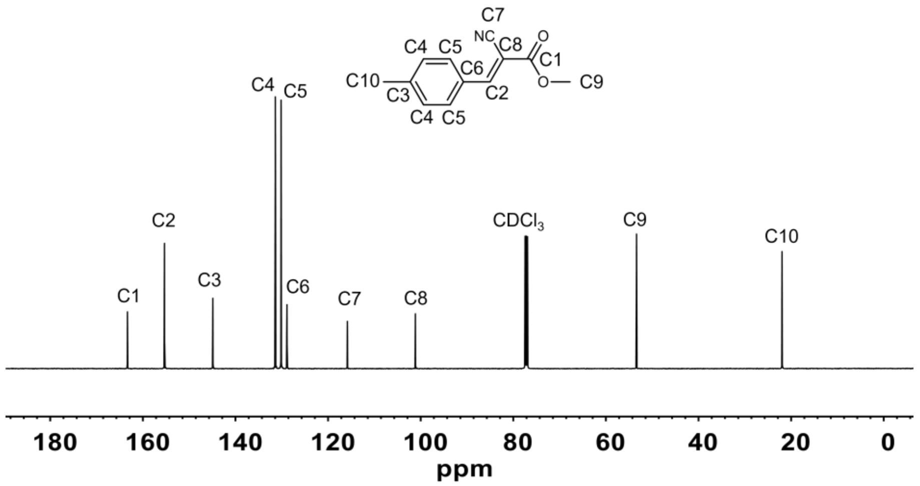

Figure S9 ^{13}C NMR (126 MHz, $CDCl_3$, 22 °C) spectrum of **M4**.

Procedures for model reactions in bulk

The Knoevenagel C=C metathesis model reaction between model compounds **M1** and **M2** to form **M1**, **M2**, **M3** and **M4** (**Figure 2a**) was performed in bulk and characterized using GC-MS and ^{1}H NMR. To avoid possible Michael addition and elimination reactions between cyanoacetate and Knoevenagel adduct[1], model compounds were recrystallized and washed repeatedly to ensure that no starting monomers or other impurities remained. In addition, to avoid hydrolysis of Knoevenagel adduct, model compounds were freeze-dried before the reaction and stored under an argon atmosphere. Organic base triazabicyclodecene (TBD, pKa: 15.2) was selected as the catalyst. Before the reaction, an equivalent amount of M1 and M2, as well as 1% mol TBD, were dissolved in anhydrous dichloromethane in vials in the glove box. After the DCM evaporated, the mixtures in vials were sealed to avoid moisture. Then the mixture was reacted at 110 °C for various durations and the difference were analyzed by GC-MS and ^{1}H NMR characterizations.

GC-MS analyses were conducted under a controlled nitrogen atmosphere, with a flow rate set at 1 mL min^{-1}. The temperature program began with an initial hold at 50 °C for 1 minute, followed by a gradual increase at a rate of 5 °C min^{-1} up to 300 °C, where it was held constant

for 5 minutes. For these analyses, samples were prepared by dissolving in dichloromethane (DCM) at a concentration of 1000 ppm.

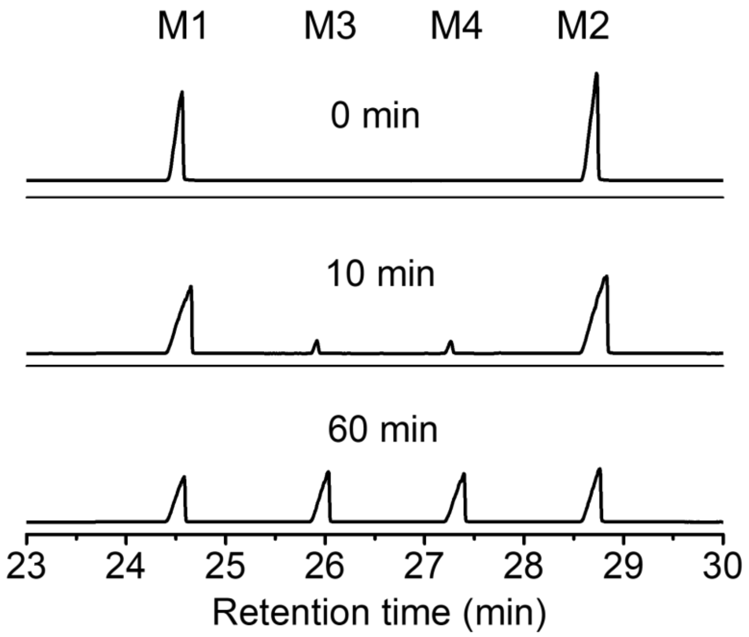

Figure S10. Gas chromatography traces illustrating the progression of the C=C metathesis reaction between an equivalent amount of **M1** and **M2** with a 1 mol% TBD as a catalyst. The spectra compare the reaction mixture before and after reaction at 110 °C for 10 and 60 minutes. The retention times for the compounds **M1**, **M2**, **M3**, and **M4** are recorded at 24.6 min, 28.8 min, 26.0 min, and 27.3 min, respectively.

For ^{1}H NMR characterization, to avoid the influence of polar solvents on the mixture, deuterated chloroform was used for ^{1}H NMR characterization of the mixture before and after the reaction. The mixture was reacted at 110 °C for different durations.

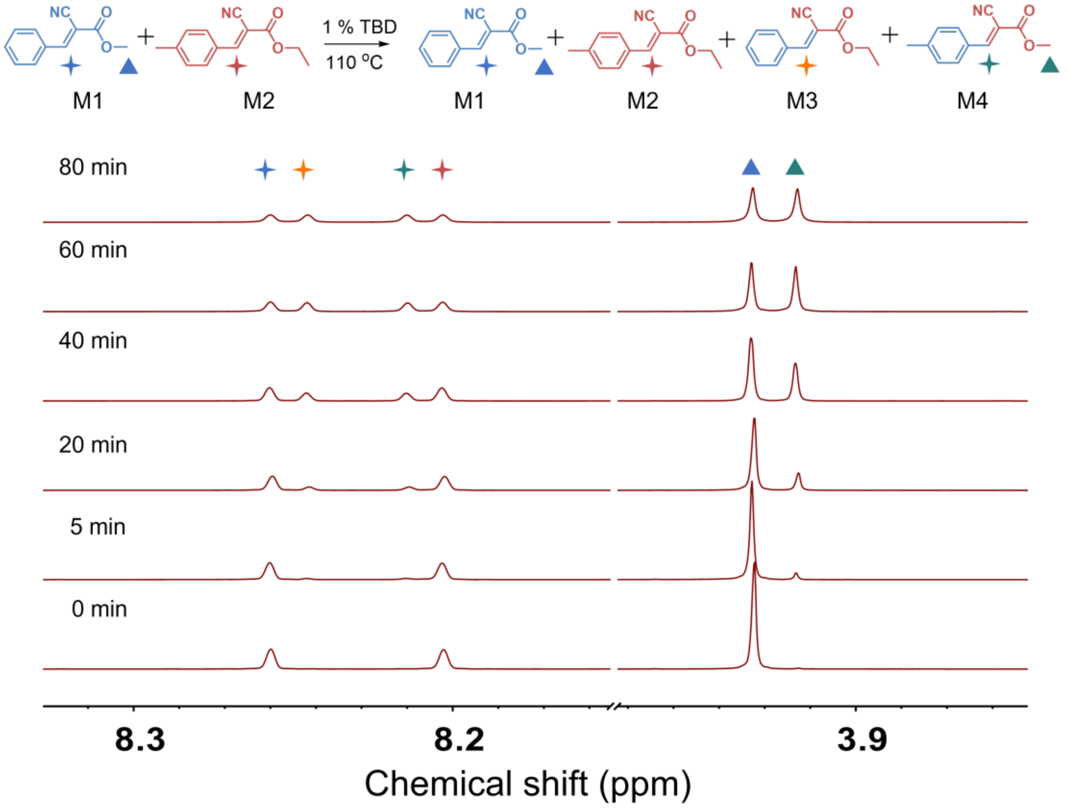

Figure S11. ^{1}H NMR (500 MHz, $CDCl_3$, 22 °C) spectra for C=C metathesis reaction model reaction between an equivalent amount of M1 and M2 at 110 °C for various durations with a 1 mol% TBD as a catalyst.

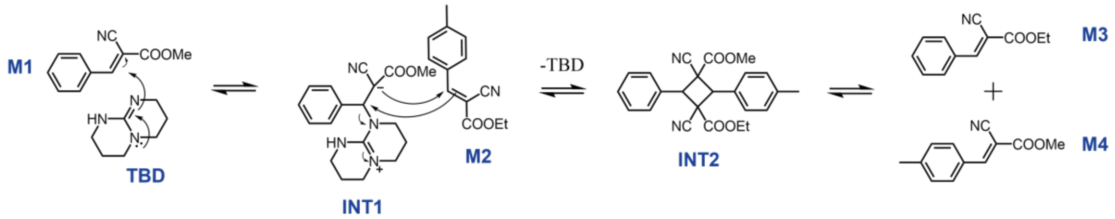

Figure S12. Proposed associative mechanism for the model C=C metathesis reaction.

Procedures for model reactions in solvents and kinetic studies

Before mixture, moisture control for model compounds and solvents was applied. Then a solution of 0.077 mol L^{-1} M1, 0.077 mol L^{-1} M2 and 0.00077 mol L^{-1} TBD (1 mol%) in $CDCl_3$ was prepared, and ^{1}H NMR experiments at various temperatures (25 °C, 30 °C 35 °C or 40 °C) were performed. The change of proton signals in -CH=C(CN)- for M1 with TMS as internal

reference were carefully integrated. The reaction progress was monitored by measuring the decrease of $[M1]/[M1]_0$ in ^{1}H NMR spectra over time (**Figure S13a**), where [M1] is the molar concentration of M1 after reaction for different times and $[M1]_0$ is the molar concentration of M1 before reaction. This reaction can be described by second-order kinetics as presented in Equation (Eq.) S1, which can be further expressed as Eq. S2, where k_{exp} is the experimental rate constant.

$$\frac{d[M1]}{dt} = -k_{exp}[M1][M2] \quad \text{(Eq. S1)}$$

$$\frac{1}{[M1]} - \frac{1}{[M1]_0} = k_{exp}(t - t_0) \quad \text{(Eq. S2)}$$

The k_{exp} under different temperatures was determined by plotting $(1/[M1]-1/[M1]_0)$ as a function of time, as shown in **Figure S13b**. The calculated values of k_{exp} are 0.00114, 0.00139, 0.00162 and 0.00226 L mol^{-1} s^{-1}for 25 ºC, 30 ºC 35 ºC and 40 ºC, respectively. The activation energy (E_a) values of the model reaction were calculated using the Arrhenius equation (Eq. S3), where B represents the fitting parameters and R is the gas constant (R = 8.314 J K^{-1} mol^{-1}). By fitting with the experimental data, E_a value was calculated to be 34 kJ mol^{-1} (**Figure S13c**).

$$\ln k_{exp} = -\frac{E_a}{RT} + B \quad \text{(Eq. S3)}$$

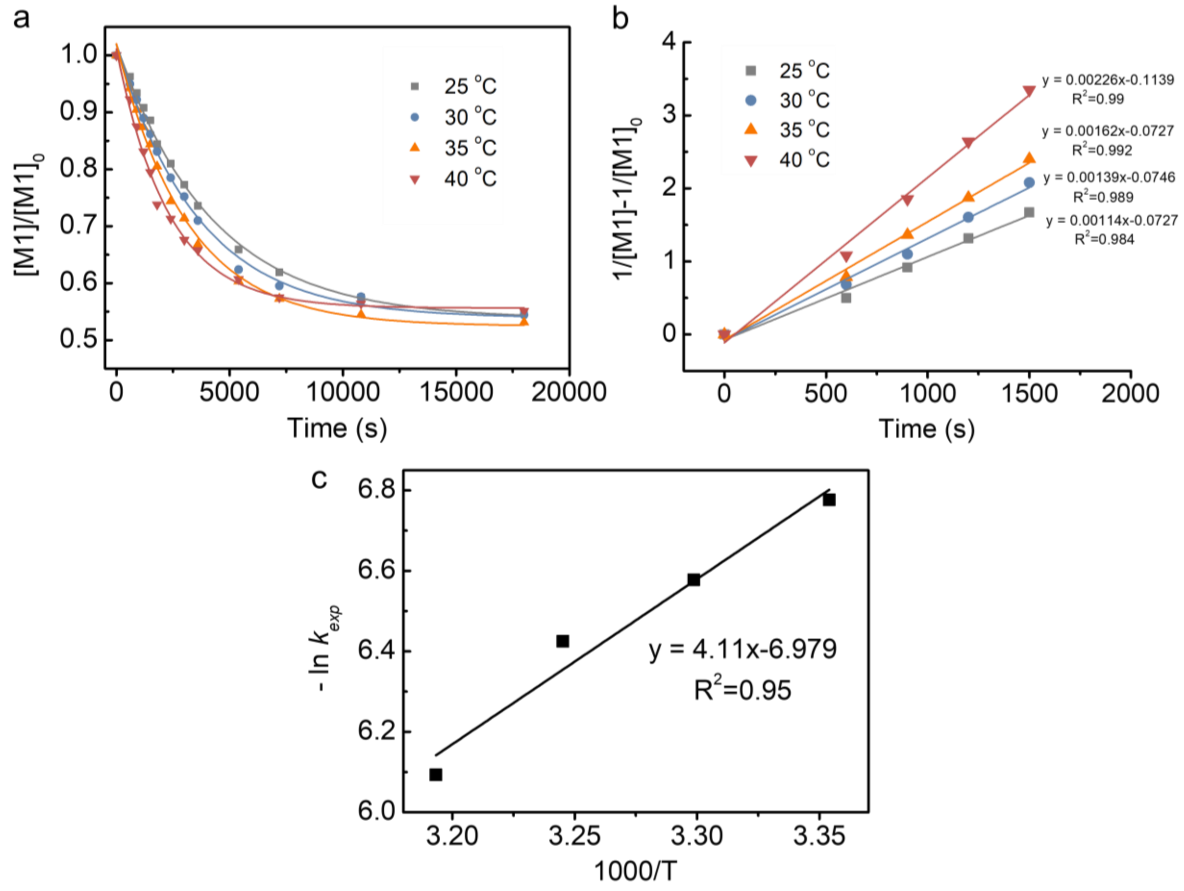

Figure S13. Kinetic studies of model reaction for M1, M2 and 1 mol% TBD in $CDCL_3$. (a) Consumption of M1 as a function of time for different temperatures; (b) Plots of (1/[M1]-1/$[M1]_0$) as a function of time for the model reaction, and k_{exp} at each temperature was determined by its slope value; (c) Arrhenius plot of experimental rate constant (-k_{exp}) vs inverse temperature (1000/T) and its linear fitting for model reaction.

In order to study the effect of catalyst nucleophilicity on the C=C metathesis reaction, triethylamine (TEA, pKa: 10.7) and N,N-diisopropylethylamine (DIPEA: 10.98) with similar pKa but distinguished nucleophilicity were selected as the catalyst. A solution of 0.077 mol L^{-1} M1, 0.077 mol L^{-1} M2 and 0.00077 mol L^{-1} TEA or DIPEA (0.2 mol%) in DMSO-d_6 were prepared, and ^1H NMR experiments at 25 °C were performed. The change of proton signals in -CH=C(CN)- for M1 with TMS as internal reference were carefully integrated and the experimental rate constants (k_{exp}) for different catalyst were calculated by using the Eq. S2 (**Figure S14**).

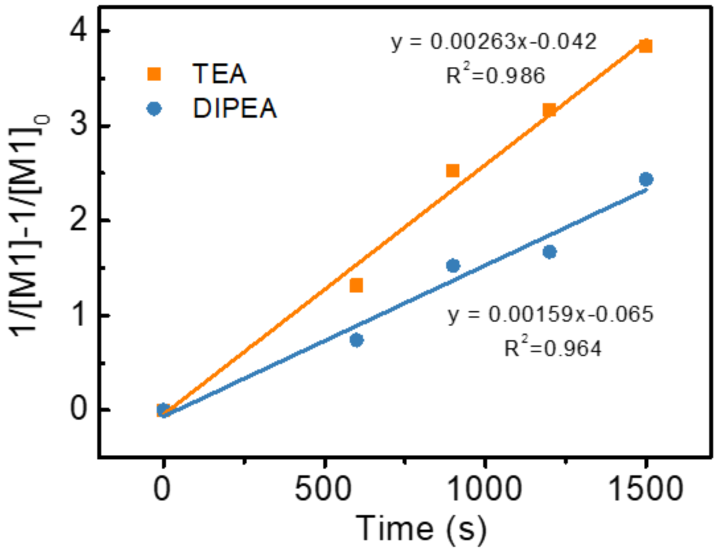

Figure S14. Plots of (1/[M1]-1/[M1]$_0$) as a function of time for the model reaction of M1, M2 and 0.2 mol% TEA or DIPEA in DMSO-d6 at 25 ºC, and k_{exp} was determined to be 0.00263 L mol^{-1} s^{-1} in the presence of TEA and 0.00159 L mol^{-1} s^{-1} under the catalysis of DIPEA.

Polymer synthesis and characterization

Synthesis of dialdehydes

The synthesis of dialdehyde compounds **4,4'-(butane-1,4-diylbis(oxy))dibenzaldehyde (A4)** and **4,4'-(octane-1,8-diylbis(oxy))dibenzaldehyde (A8)** were performed using the same procedures. Take the synthesis of A4 as an example: A mixture of 4-hydroxybenzaldehyde (23.1 g, 189.0 mmol) and potassium carbonate (26.1 g, 189.0 mmol) in anhydrous DMF (100 mL) was prepared and heated to 60 °C. Subsequently, a solution of 1,4-dichlorobutane (10 g, 78.7 mmol) in anhydrous DMF (20 mL) was added dropwise into the mixture. The resulting mixture was then stirred at 90 °C for 12 hours. Afterward, the solution was allowed to cool and was poured into deionized water while vigorously stirring to induce precipitation. The crude product was collected via vacuum filtration, washed sequentially with water and isopropanol, and then subjected to vacuum drying at 70 °C for 24 hours to obtain dialdehyde compound A4.

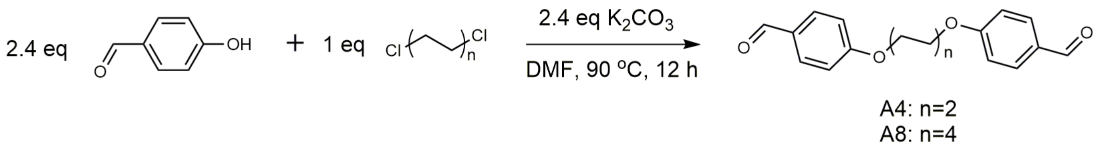

Figure S15. General synthetic route for the synthesis of dialdehyde compounds **A4** and **A8**.

4,4'-(butane-1,4-diylbis(oxy))dibenzaldehyde (A4): ^{1}H NMR (500 MHz, Chloroform-*d*) δ 9.85 (s, 2H), 7.81 (d, *J* = 8.3 Hz, 4H), 6.96 (d, *J* = 10.5 Hz, 4H), 4.11 (s, 4H), 2.01 (s, 4H). ^{13}C NMR (126 MHz, Chloroform-*d*) δ 190.86, 164.06, 132.08, 130.00, 114.82, 67.86, 25.86. MS (m/z): [M] calculated for $C_{18}H_{18}O_4$, 298.1205; found 298.1430.

4,4'-(octane-1,8-diylbis(oxy))dibenzaldehyde (A8): ^{1}H NMR (500 MHz, Chloroform-*d*) δ 9.87 (s, 2H), 7.81 (s, 4H), 6.98 (s, 4H), 4.03 (t, *J* = 6.4 Hz, 4H), 1.91 – 1.71 (m, 4H), 1.48 (d, *J* = 9.0 Hz, 4H), 1.41 (s, 4H). ^{13}C NMR (126 MHz, Chloroform-*d*) δ 190.97, 164.39, 132.16, 129.94, 114.91, 68.51, 29.40, 29.20, 26.08. MS (m/z): [M] calculated for $C_{22}H_{26}O_4$, 358.1831; found, 358.2270.

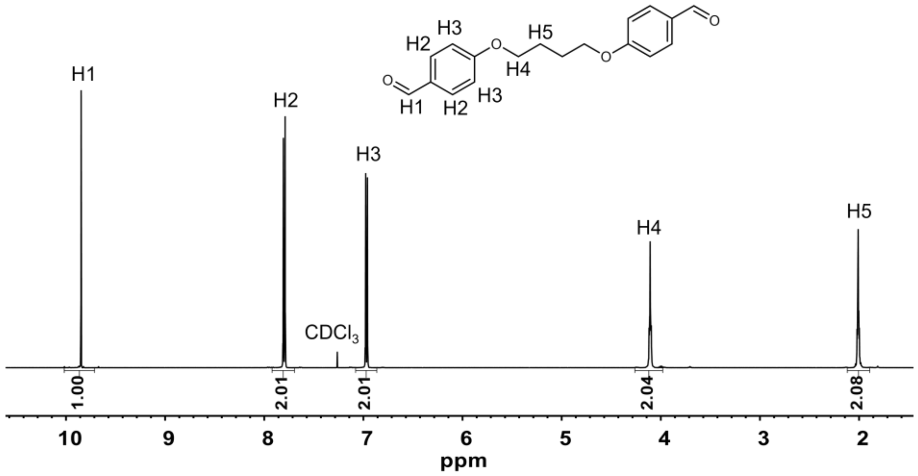

Figure S16. ^{1}H NMR (500 MHz, $CDCl_3$, 22 °C) spectrum of **A4**.

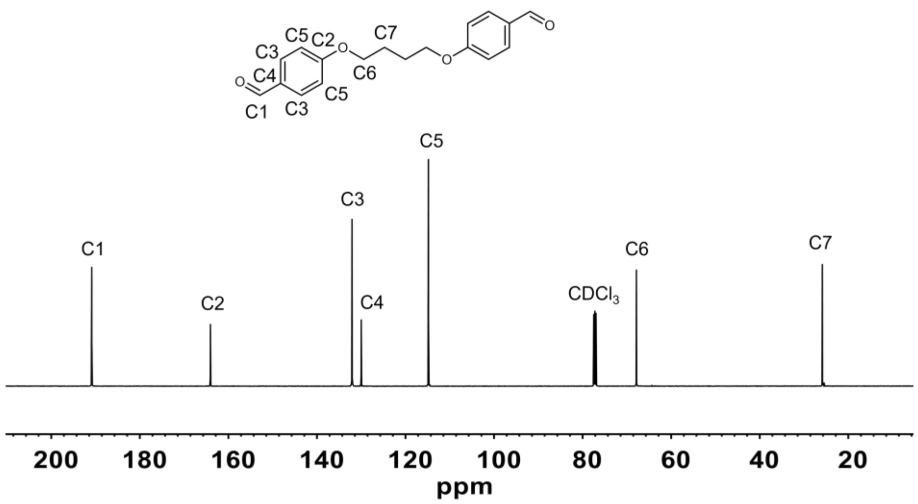

Figure S17. ^{13}C NMR (126 MHz, $CDCl_3$, 22 °C) spectrum of **A4**.

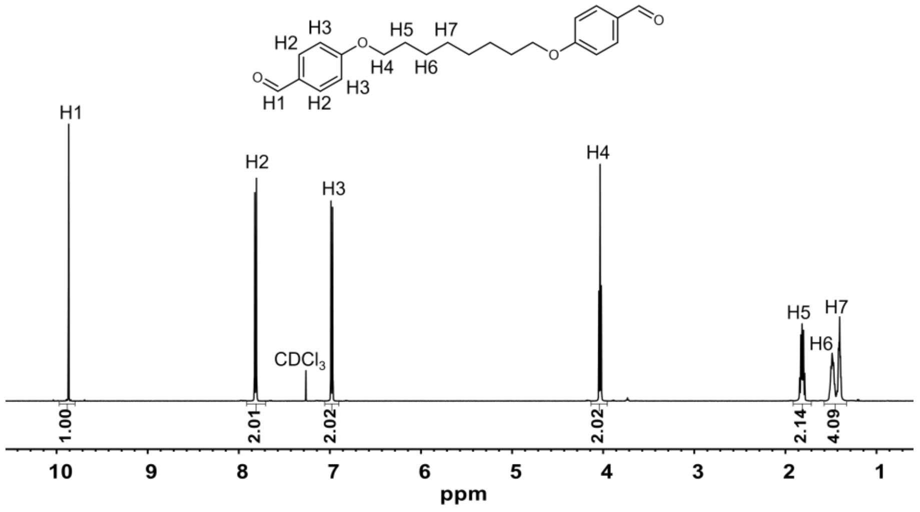

Figure S18. ^{1}H NMR (500 MHz, $CDCl_3$, 22 °C) spectrum of **A8**.

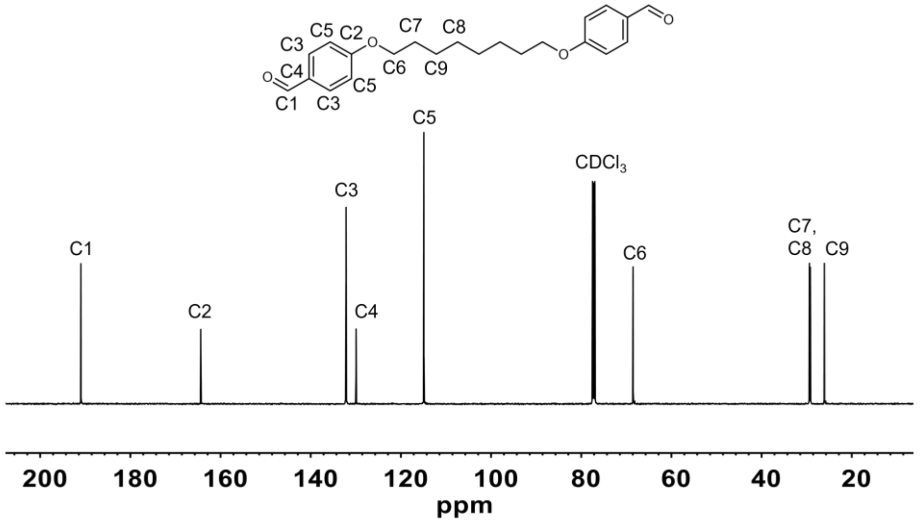

Figure S19. ^{13}C NMR (126 MHz, $CDCl_3$, 22 °C) spectrum of **A8**.

Synthesis of 4,4'-(((ethane-1,2-diylbis(oxy))bis(ethane-2,1-diyl))bis(oxy))dibenzaldehyde

(B8): A mixture of 4-hydroxybenzaldehyde (19.6 g, 160.5 mmol) and potassium carbonate (22.2 g, 160.5 mmol) in anhydrous DMF (100 mL) was prepared and heated to 60°C. Subsequently, a solution of 1,2-Bis(2-chloroethoxy)ethane (10 g, 53.5 mmol) in anhydrous DMF (20 mL) was added dropwise into the mixture. The resulting mixture was then stirred at 90°C for 12 hours. Following cooling, DCM was introduced into the solution and stirred for 10 minutes. Insoluble solids were removed by filtration, and the solution was followed by triple extraction with DI water. The DCM solution was then concentrated by rotary evaporation, and the resulting solid was precipitated into isopropanol. After vacuum filtration and a subsequent isopropanol wash, the powder was dried under vacuum at 70°C for 24 hours to yield the dialdehyde compound B8.

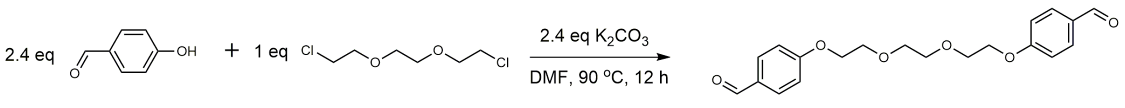

Figure S20. Synthetic route of the dialdehyde compound **B8.**

4,4'-(((ethane-1,2-diylbis(oxy))bis(ethane-2,1-diyl))bis(oxy))dibenzaldehyde (B8): ^{1}H NMR (500 MHz, Chloroform-*d*) δ 9.84 (s, 1H), 7.79 (d, J = 7.5 Hz, 2H), 6.98 (s, 2H), 4.17 (s, 2H), 3.86 (s, 2H), 3.73 (s, 2H). ^{13}C NMR (126 MHz, Chloroform-*d*) δ 190.88, 163.90, 132.02, 130.13, 114.96, 71.00, 69.61, 67.83. MS (m/z): [M] calculated for $C_{20}H_{22}O_6$, 358.1416; found, 358.1665.

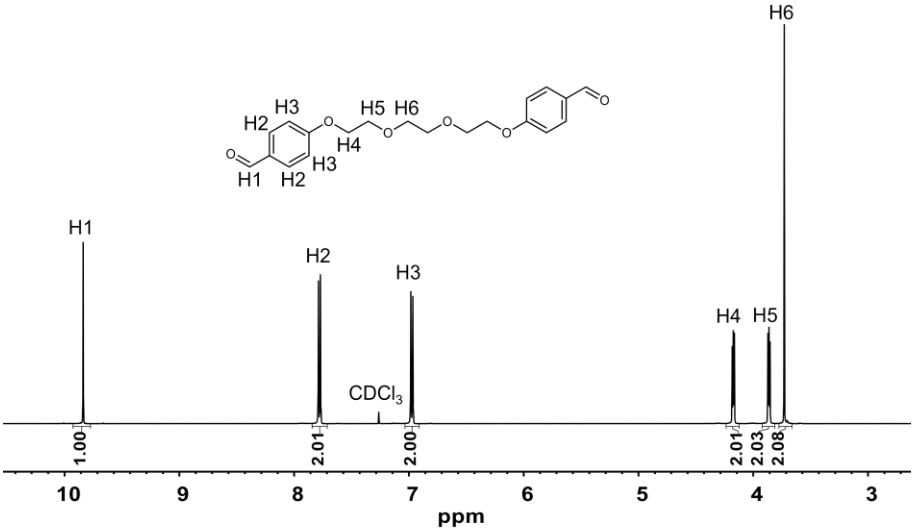

Figure S21. ^{1}H NMR (500 MHz, $CDCl_3$, 22 °C) spectrum of **B8**.

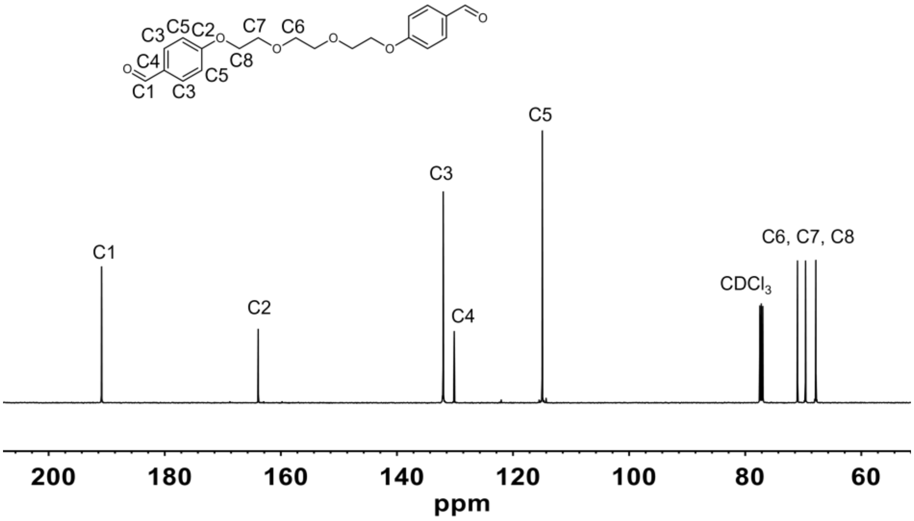

Figure S22. ^{13}C NMR (126 MHz, $CDCl_3$, 22 °C) spectrum of **B8**.

Synthesis of a tri-arm cyanoacetate (TCA)

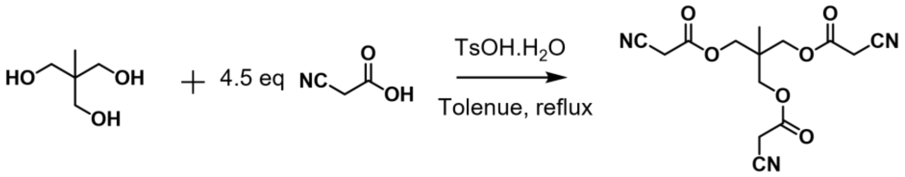

Figure S23. Synthetic route of a tri-arm cyanoacetate (**TCA**).

A tri-arm cyanoacetate (TCA) was synthesized by esterification reaction between trimethylolethane and cyanoacetic acid by using the method reported in our previous work[1].

Preparation of poly(α-cyanocinnamate)s (PCCs).

PCCs were synthesized from TCA and a dialdehyde compound (A4, A8, or B8) through Knoevenagel polycondensation with TBD as a catalyst (**Table S1**). Except for the use of different dialdehyde compounds, the other curing conditions are the same. Here we take the synthesis of PCC-A4 as an example. A mixture of TCA (1 g, 3.12 mmol) and A4 (1.39 g, 4.67 mmol) was dissolved in THF (6 mL) by stirring at RT, followed by adding the catalyst TBD (0.065 g, 0.47 mmol). The solution was then transferred into a polytetrafluoroethylene (PTFE) petri dish with a diameter of 8 cm. The solvent was allowed to slowly evaporate at a fume hood for 3 h. The obtained film was pre-cured at 60 °C for 30 min under vacuum and post-cured using a hot press (Collin P 200 PM) under 30 bar pressure at 170 °C for 30 min. After annealing, a pale yellow and transparent PCC-A4 film with a thickness of approximately 150 um was obtained (**Figure S24**).

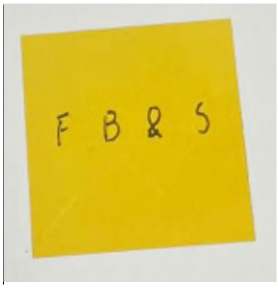

Figure S24. Digital photograph displaying a defect-free and transparent PCC-A4 film

Structural characterization

The curing process between a dialdehyde and TCA was monitored using UV-vis spectroscopy. TCA (1 eq), A4 (1.5 eq) and TBD (0.15 eq) were dissolved in THF at a concentration of 1 wt. %. After a period of 30 minutes, the solution was carefully drip-coated onto a quartz sheet. Following the evaporation of the solvent THF in a fume hood at RT, a transparent and continuous thin layer was formed on the quartz sheet. Subsequently, the coated quartz sheet was subjected to a stepwise temperature increase from 60 °C to 180 °C under vacuum. The sample at a specific temperature was heated for 30 minutes, and UV-vis tests were performed before and after each heating cycle. The temperature-dependent UV-vis absorbance spectra are presented in **Figure 3b**.

FTIR spectroscopy was used to analyze the structure of the starting monomers and cured polymers. Solid state spectra were recorded in attenuated total reflectance (ATR) mode and converted to absorbance spectra. The results are displayed in **Figure S25**.

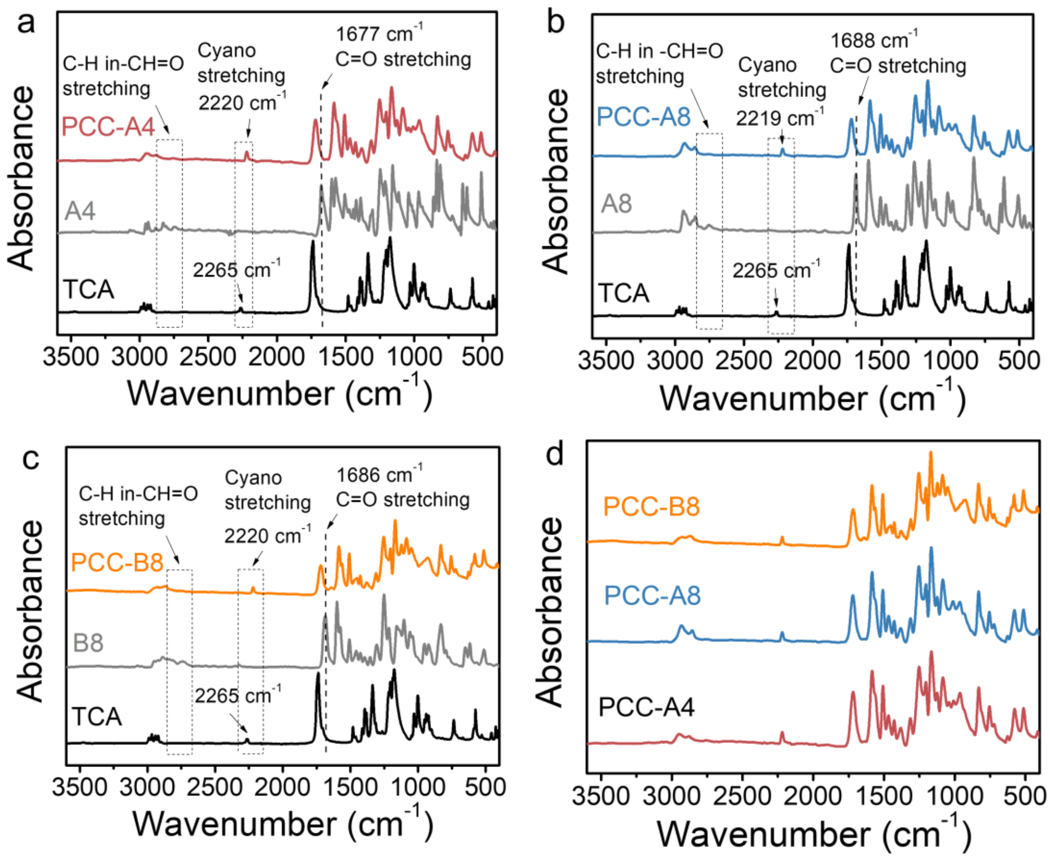

Figure S25. FT-IR spectra of (a) TCA, A4 and PCC-A4; (b) TCA, A8 and PCC-A8; (c) TCA, B8 and PCC-B8; (d) PCC-A4, PCC-A8 and PCC-B8.

Swelling ratio and gel fraction tests.

Approximately 200 mg PCC samples were submerged in 20 mL vials containing 15 mL of various solvents, including tetrahydrofuran (THF), ethanol (EtOH), chloroform and acetone. The samples were then kept at 25 °C for two days. Afterward, the solvents were decanted, and the residues were washed five times with the same solvents before being dried in a vacuum oven at 100 °C for 24 hours to ensure complete solvent removal. The swelling ratio and gel fraction (%) were calculated using Eq. S4 and S5, respectively. In these equations, m_0 represents the original mass of the sample before testing, m_1 is the mass of the swollen sample, and m_2 is the final mass after drying.

$$Swelling\ ratio = 100\% * \frac{m_1 - m_0}{m_0} \qquad \text{(Eq. S4)}$$

$$Gel\ fraction\ = 100\% * \frac{m_1}{m_0} \quad \text{(Eq. S5)}$$

To demonstrate solvent stability, rectangular samples of PCCs were soaked in 4 ml THF for 7 days. Their appearances before and after soaking were recorded in **Figure S26**.

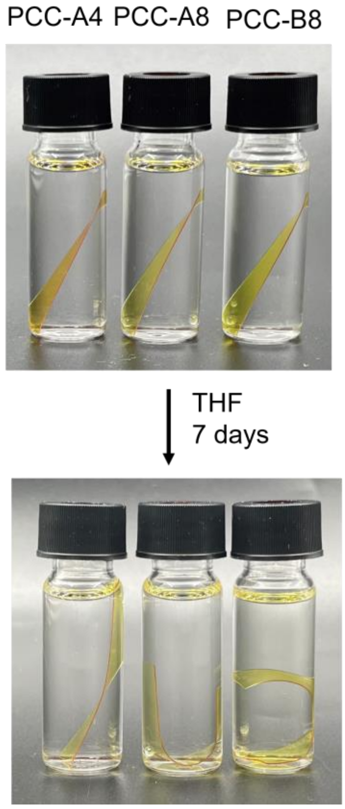

Figure S26. The images of PCCs before and after immersion in THF (4 ml) for 7 days.

Differential scanning calorimetry (DSC) tests.

PCC samples were loaded into aluminum pans and scanned against an empty reference pan. The temperature was ramped up at 20 °C min^{-1} from 25 °C to 180 °C and kept at 180 °C for 2 min, followed by being cooled to 25 °C at a cooling rate of 20 °C min^{-1}, and then heated to 200 °C at a heating rate of 10 °C min^{-1}. The heating curves obtained from the second ramp were used for analysis purposes. Glass transition temperature (T_g) was found by taking the midpoint of the reversible endotherm of the second heating curve for each sample.

Dynamic mechanical analysis (DMA) tests.

The rectangular samples with dimensions of approximately approximately 20 mm (length) x 5 mm (width) x 0.15 mm (thickness) were equilibrated at 30 °C for 5 minutes and then heated at a rate of 3 °C min^{-1} at an oscillation frequency of 1 Hz.

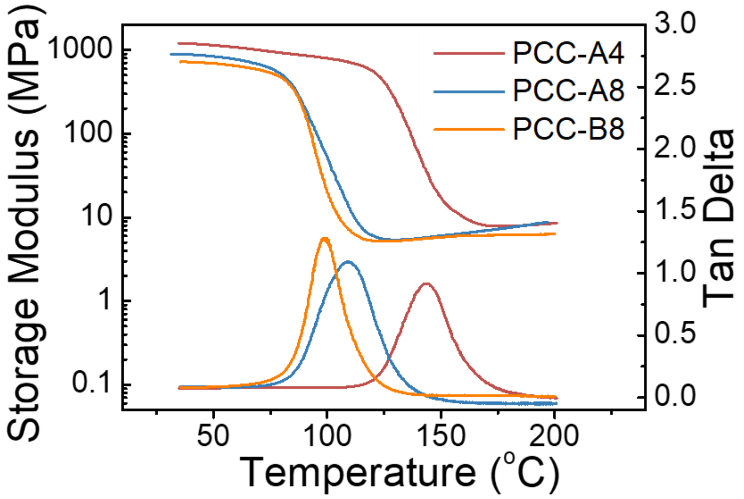

Figure S27. Storage modulus and tan δ as a function of temperature for the PCCs tested by DMA temperature sweep.

Calculation of the molecular weight between crosslinks (M_c)

M_c was calculated using the tensile storage modulus (E′) from the rubbery plateau of storage modulus curves, as shown in Eq. S6[2].

$$G' = \frac{E'}{2(1+v)} = \frac{\rho RT}{M_c} \qquad \text{Eq. S6}$$

$$M_c = \frac{3\rho RT}{E'}$$

The density (ρ) of PCC-A4, PCC-A8 and PCC-B8 were measured to be 1480, 1420 and 1400 kg m^{-3}, respectively. Poisson's ratio (v = 0.4) of Rubber was used. R is the gas constant (R = 8.314 J/mol*K). E′ is the plateau modulus in the rubbery state at T_g + 40 °C (Pa), T is the Kelvin temperature of T_g + 40 °C. The calculated M_c was 2116, 2559, 2623 g mol^{-1} for PCC-A4, PCC-A8 and PCC-B8, respectively.

Thermogravimetric analysis (TGA) tests.

Samples (around 5 mg) were tested from 50 to 700 °C with a heating rate of 10 °C min^{-1} under a nitrogen atmosphere.

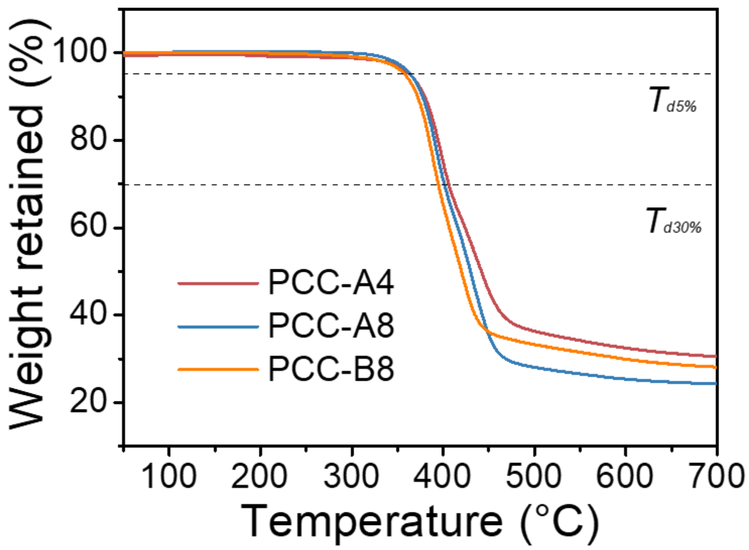

Figure S28. TGA curves of PCCs under a nitrogen atmosphere.

Tensile tests and FESEM imaging of tensile cross-section

The PCC films were cut into rectangular strips with dimensions of approximately 30 mm (length) x 5 mm (width) x 0.15 mm (thickness). Tensile tests were conducted at RT with a strain rate at 5% min^{-1} until fracture. The tensile properties of each sample were determined by taking the average of three measurements.

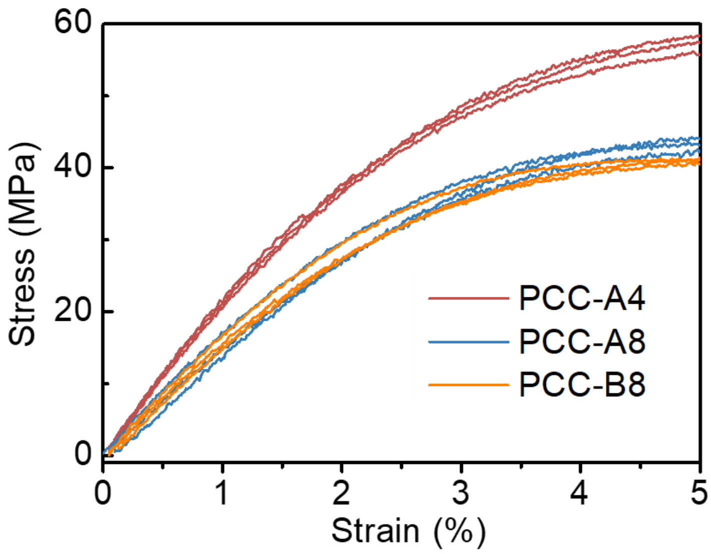

Figure S29. Partially enlarged stress-strain diagrams (strain ranges from 0 to 5%) of the developed PCCs to facilitate distinguishing Young's modulus differences.

The micromorphology of tensile cross-section for differene samples was imaged by FESEM. Before imaging, the samples were coated with a thin layer of gold using a gold sputtering equipment (Jeol JFC-1200).

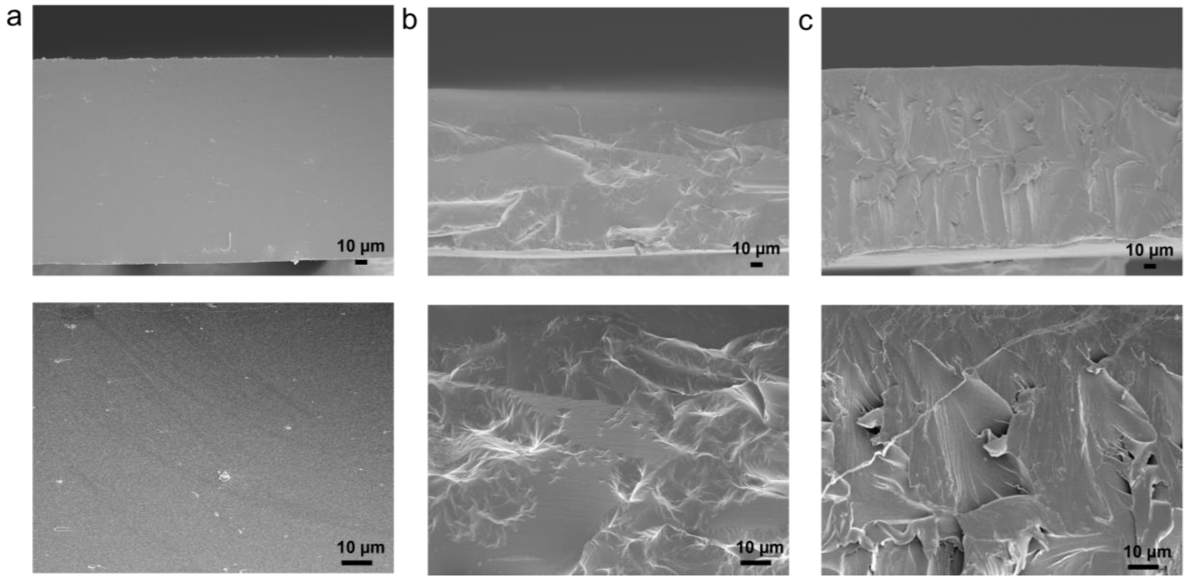

Figure S30. FESEM images of tensile cross-section of (a) PCC-A4, (b) PCC-A8, and (c) PCC-B8.

Malleability and reprocessability of PCC

Stress relaxation measurements and activation energy calculation

The rectangular sample was initially preloaded by 1×10^{-3} N force to maintain straightness and equilibrated at the set temperature for 5 min before being stretched by 2% strain on a DMA machine. The deformation was maintained throughout the test and the decrease of the stress relaxation modulus was recorded. The relaxation time refers to the time at which the relaxation modulus decreases to 1/e (~36.7 %) at the set temperatures. The activation energy (E_a) of the bond exchange within bulk polymers was calculated via the Arrhenius equation (Eq. S7)[3].

$$\tau^*(T) = \tau_0 \exp(E_a/RT) \quad \text{(Eq. S7)}$$

Where the relaxation time τ^* was determined via modulus relaxation to 1/e. τ_0 is the characteristic relaxation time at infinite temperature, T is the experimental temperature, and R is the universal gas constant.

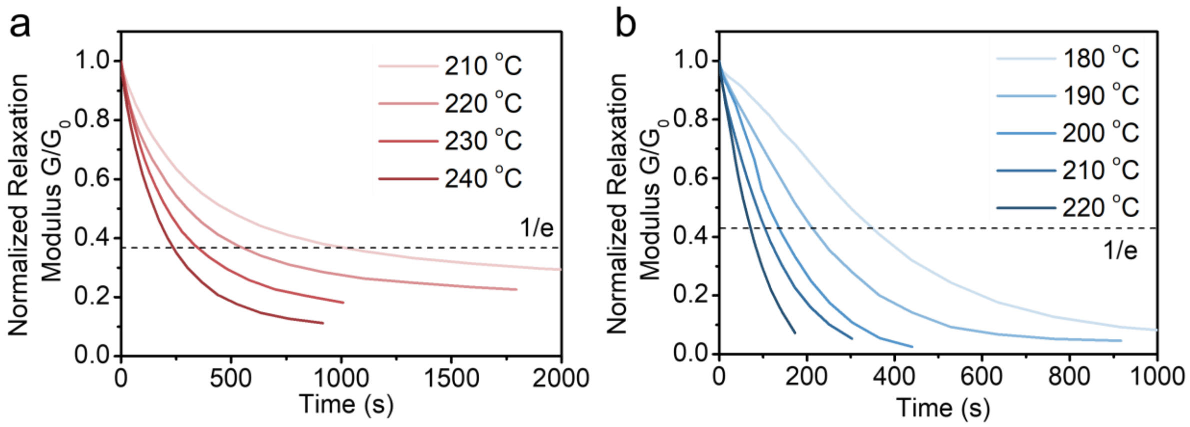

Figure S31. Normalized stress relaxation curves of (a) PCC-A4 and (b) PCC-A8 at various temperatures.

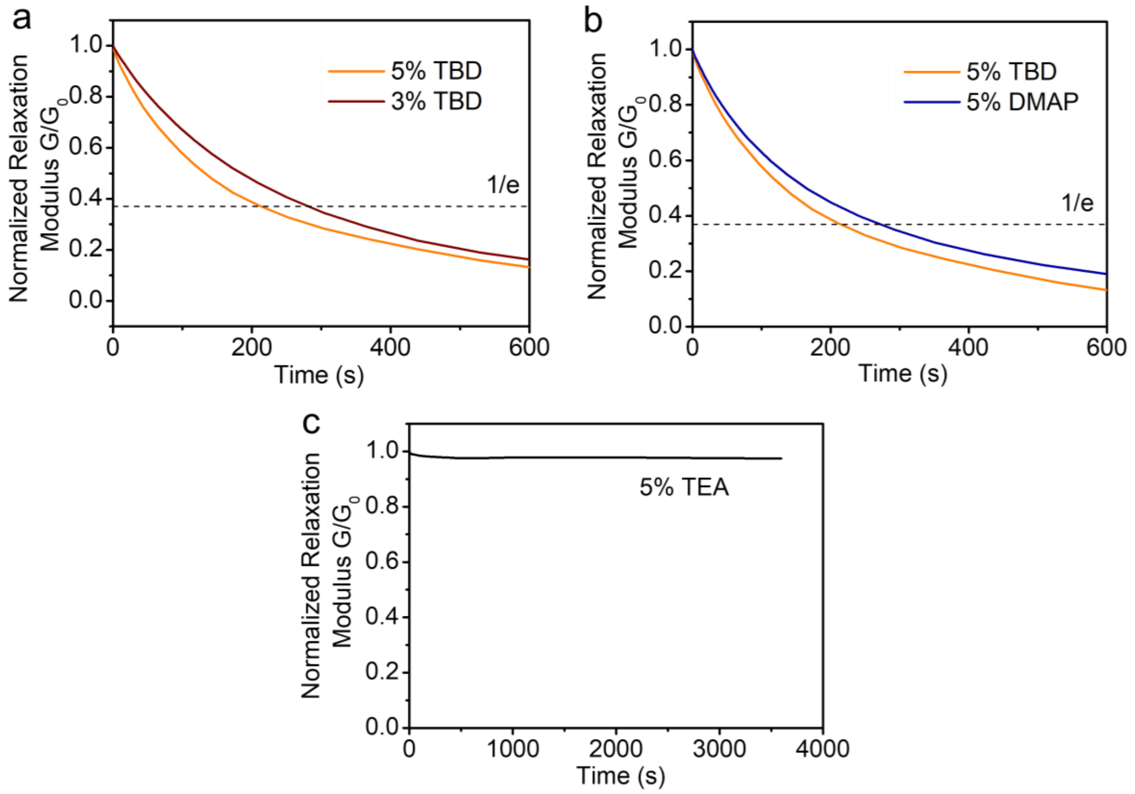

Figure S32. Normalized stress relaxation curves of PCC-B8 with (a) 3 mol% or 5 mol% TBD, (b) 5 mol% TBD or DMAP, and (c) 5 mol% TEA as a catalyst at 160 °C.

Determination of the topology freezing temperature (T_v)

T_v is defined as the temperature at which a material transitions from a viscoelastic solid to a viscoelastic liquid. This transition is typically identified when the viscosity of the material reaches 10^6 MPa s. The viscosity can be calculated from DMA temperature sweep tests and stress relaxation experiments by using the Maxwell equation (Eq. S8)[4]:

$$\eta = G'\tau = \frac{E'}{2(1+v)} \qquad \text{Eq. S8}$$

$$\eta = 10^6 \ \text{MPa s} = \frac{E'}{2(1+v)}\tau$$

E' is the plateau modulus in the rubbery state at T_g + 40 °C (MPa), the relaxation time (τ) can be calculated when the viscosity reaches 10^6 MPa s. The calculated τ values for PCC-A4, PCC-A8 and PCC-B8 are $3.77*10^5$ s, $5.14*10^5$ s and $5.46*10^5$ s, respectively. T_v can be calculated from the relaxation time (τ) via the Arrhenius relationship (Eq. S7).

Reprocessing process

The PCC-B8 film was cut into small pieces and sandwiched between two steel sheets covered with PTFE films. The assembly was subjected to hot pressing at 160 °C under a pressure of 30 bar for 10 min. After cooling to RT, a defect-free and transparent film (**Figure 5c**) was obtained. The same procedures were followed for 2nd cycle reprocessing. The 1st and 2nd cycles reprocessed samples were named PCC-B8-R1 and PCC-B8-R2, respectively. To assess the properties of the reprocessed PCCs, FT-IR, DMA and tensile tests were conducted with same conditions as the original one.

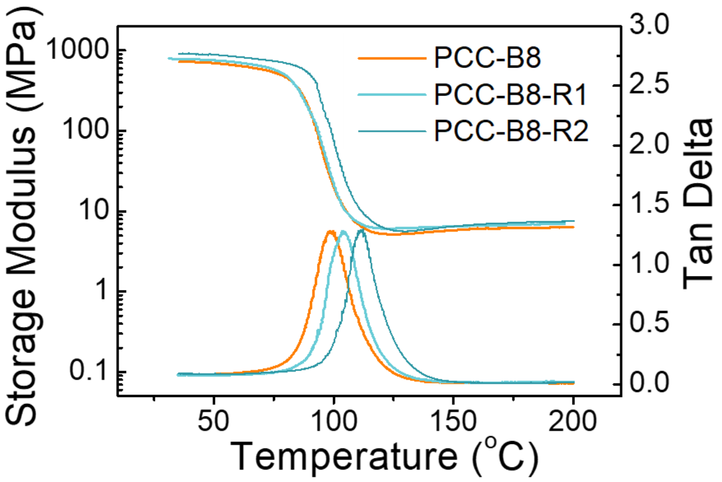

Figure S33. Storage modulus and tan delta curves of original and 2 cycles reprocessed PCC-B8 tested by DMA temperature sweep.

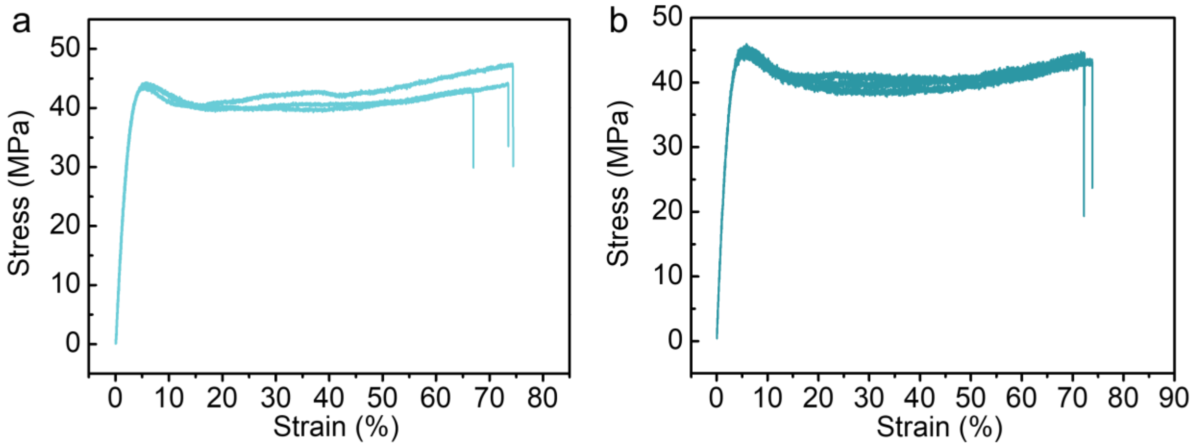

Figure S34. Stress-strain curves of (a) 1st cycle and (b) 2nd cycle reprocessed PCC-B8.

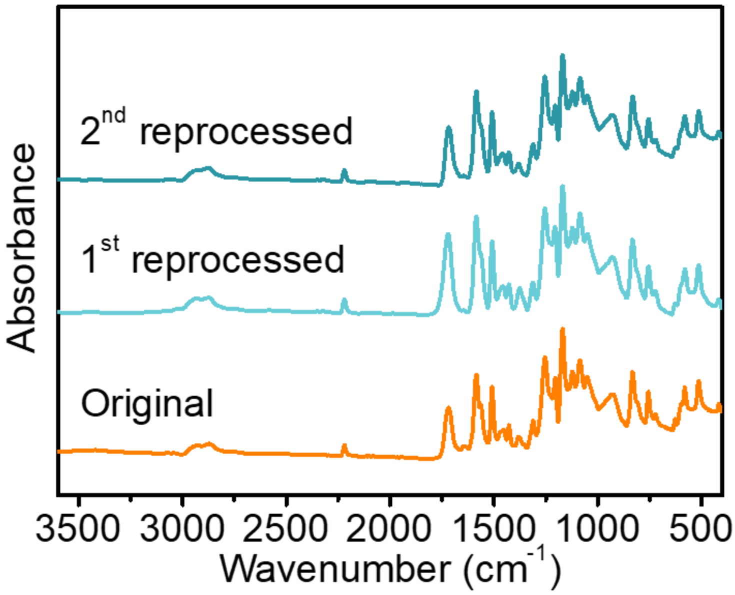

Figure S35. FTIR spectra of original and two cycles reprocessed PCC-B8.

Supporting Tables

Table S1. Composition of TCA and dialdehydes for synthesis of PCCs.

Samples	TCA	A4	A8	B8	TBD
PCC-A4	1 eq	1.5 eq			0.15 eq
PCC-A8	1 eq		1.5 eq		0.15 eq
PCC-B8	1 eq			1.5 eq	0.15 eq

Table S2. Swelling ratio and gel fraction test results for different PCCs.

Sample	Solvent	Swelling ratio (%)	Gel fraction (%)
PCC-A4	THF	58	99.8
	EtOH	0	100
	Chloroform	110	97.7
	Acetone	13	98.4
PCC-A8	THF	73	99.3
	EtOH	0	100
	Chloroform	147	96.2
	Acetone	24.8	97.2
PCC-B8	THF	82	97.7
	EtOH	1.5	98.8
	Chloroform	180	97.1
	Acetone	25.9	96.6

Table S3. Thermal and mechanical properties, alongside solid-state dynamics of PCCs, previously reported vitrimers based on different dynamic covalent bonds and traditional thermosets.

Vitrimers based on different dynamic covalent bonds or types of **traditional thermosets**	Polymer performance				Solid-state bond exchange		References
	T_g (°C)	E (MPa)	σ (MPa)	ε (%)	E_a (kJ mol^{-1})	T_v (°C)	
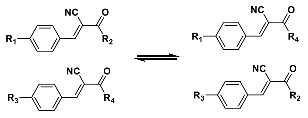	134.6	2280±41	62±0.4	17.7±1.9	99.7	117	PCC-A4
	102.2	1530±112	45±0.7	25.9±0.8	71.9	56	PCC-A8
	90.9	1590±39	43±1	79.1±1.9	66.9	33	PCC-B8
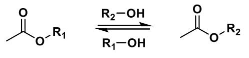	80	1800	55	~ 8	88	53-165	[3]
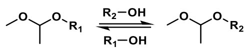	66–71	800-1100	27.2–33.3	5	126–136	89-99	[5]
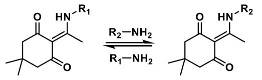	125	1800	37	5	49-60	-	[6]
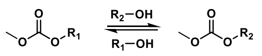	15-35	100-1200	7-36	4	79-123	-	[7]
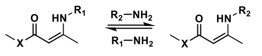 X = CH_2, O or NH	87	2400	91	~6.2	60	29	[8]
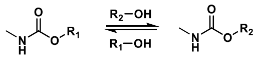	54	2200	72	6.9	111-148	111	[9]
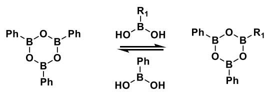	~65	559-768	17.8-32.9	5.6-13.7	79.5	-0.5	[10]
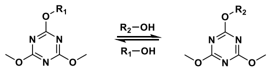	27.4	2.93	2.64	118	76.7	-	[11]
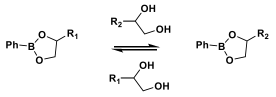	-	3.23-4.86	1.12-1.97	121-446	-	-	[12]
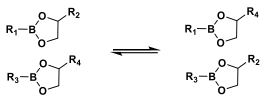	-	~500	~19	~700	43.2-76.7	-	[13]
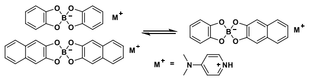	87-114	1130-1510	32-35.5	3.8-5.4	-	-	[14]
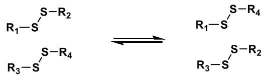	130	2600	88	7.1	55	-13	[15]
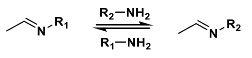	18-135	130-1000	10-64	5-150	33.5-129	-	[16]
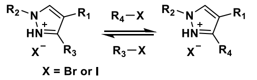 X = Br or I	-11	8	1.2	~22	140	98	[17]
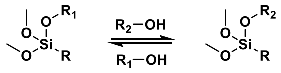	~125	1300-1667	11.9-18.7	15.7-16	81-174	47-117	[4]

Vitrimers based on different dynamic covalent bonds or types of **traditional thermosets**	Polymer performance				Solid-state bond exchange		References
	T_g (°C)	E (MPa)	σ (MPa)	ε (%)	E_a (kJ mol^{-1})	T_v (°C)	
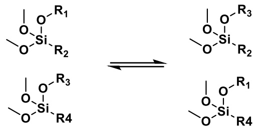	-125	101	8.2	180	77.8	45	18
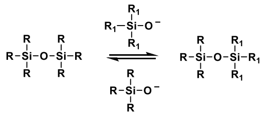	83	2200	46.6	4	40	-43	19
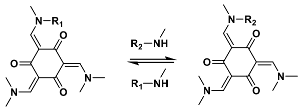	120	571	79.4	13.9	91.4	-	20
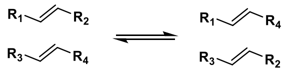	-	0.94–1.1	-	-	-	-	21
**Bisphenol A epoxy resin**	164	2779	87	5.9	-	-	22
**Melamine formaldehyde**	-	-	45-52	-	-	-	23
**Phenol formaldehyde**	170	3800	48	2	-	-	23
**Thermoset polyimide**	320	1700	79.1	3.1	-	-	24
**Thermoset polyurethane**	130	1717	74	8	-	-	25
**Unsaturated polyester**	57.5	1065	52.7	7	-	-	26

T_g: glass transition temperature; E: Young's modulus; σ: ultimate tensile strength; ε: strain at the break; E_a: activation energy for solid-state bond exchange within polymer networks; T_v: topology freezing transition temperature.

Refrences

1 Wang, S. *et al.* Recyclable, Malleable, and Strong Thermosets Enabled by Knoevenagel Adducts. *J. Am. Chem. Soc.* **146**, 9920–9927 (2024).

2 Lessard, J. J. *et al.* Catalyst-free vitrimers from vinyl polymers. *Macromolecules* **52**, 2105-2111 (2019).

3 Montarnal, D., Capelot, M., Tournilhac, F. & Leibler, L. Silica-like malleable materials from permanent organic networks. *Science* **334**, 965-968 (2011).

4 Nishimura, Y., Chung, J., Muradyan, H. & Guan, Z. Silyl ether as a robust and thermally stable dynamic covalent motif for malleable polymer design. *J. Am. Chem. Soc.* **139**, 14881-14884 (2017).

5 Li, Q. *et al.* Facile catalyst-free synthesis, exchanging, and hydrolysis of an acetal motif for dynamic covalent networks. *J. Mater. Chem. A* **7**, 18039-18049 (2019).

6 Christensen, P. R., Scheuermann, A. M., Loeffler, K. E. & Helms, B. A. Closed-loop recycling of plastics enabled by dynamic covalent diketoenamine bonds. *Nat. Chem.* **11**, 442-448 (2019).

7 Snyder, R. L., Fortman, D. J., De Hoe, G. X., Hillmyer, M. A. & Dichtel, W. R. Reprocessable acid-degradable polycarbonate vitrimers. *Macromolecules* **51**, 389-397 (2018).

8 Denissen, W. *et al.* Vinylogous urethane vitrimers. *Adv. Funct. Mater.* **25**, 2451-2457 (2015).

9 Fortman, D. J., Brutman, J. P., Cramer, C. J., Hillmyer, M. A. & Dichtel, W. R. Mechanically activated, catalyst-free polyhydroxyurethane vitrimers. *J. Am. Chem. Soc.* **137**, 14019-14022 (2015).

10 Ogden, W. A. & Guan, Z. Recyclable, strong, and highly malleable thermosets based on boroxine networks. *J. Am. Chem. Soc.* **140**, 6217-6220 (2018).

11 Lei, Z. *et al.* Recyclable and malleable thermosets enabled by activating dormant dynamic linkages. *Nat. Chem.* **14**, 1399-1404 (2022).

12 Cromwell, O. R., Chung, J. & Guan, Z. Malleable and self-healing covalent polymer networks through tunable dynamic boronic ester bonds. *J. Am. Chem. Soc.* **137**, 6492-6495 (2015).

13 Röttger, M. *et al.* High-performance vitrimers from commodity thermoplastics through dioxaborolane metathesis. *Science* **356**, 62-65 (2017).

14 Chen, H. *et al.* Spiroborate-Linked Ionic Covalent Adaptable Networks with Rapid Reprocessability and Closed-Loop Recyclability. *J. Am. Chem. Soc.* **145**, 9112-9117 (2023).

15 de Luzuriaga, A. R. *et al.* Epoxy resin with exchangeable disulfide crosslinks to obtain reprocessable, repairable and recyclable fiber-reinforced thermoset composites. *Mater. Horiz.* **3**, 241-247 (2016).

16 Taynton, P. *et al.* Repairable woven carbon fiber composites with full recyclability enabled by malleable polyimine networks. *Adv. Mater.* **28**, 2904-2909 (2016).

17 Obadia, M. M., Mudraboyina, B. P., Serghei, A., Montarnal, D. & Drockenmuller, E. Reprocessing and recycling of highly cross-linked ion-conducting networks through transalkylation exchanges of C–N bonds. *J. Am. Chem. Soc.* **137**, 6078-6083 (2015).

18 Tretbar, C. A., Neal, J. A. & Guan, Z. Direct silyl ether metathesis for vitrimers with exceptional thermal stability. *J. Am. Chem. Soc.* **141**, 16595-16599 (2019).

19 Wu, X. *et al.* A facile access to stiff epoxy vitrimers with excellent mechanical properties via siloxane equilibration. *J. Mater. Chem. A* **6**, 10184-10188 (2018).

20 Hu, Z. *et al.* Reprocessible Triketoenamine- Based Vitrimers with Closed- Loop Recyclability. *Angew. Chem. Int. Ed.* **62**, e202306039 (2023).

21 Lu, Y.-X., Tournilhac, F., Leibler, L. & Guan, Z. Making insoluble polymer networks malleable via olefin metathesis. *J. Am. Chem. Soc.* **134**, 8424-8427 (2012).

22 Ma, S. *et al.* Readily recyclable, high-performance thermosetting materials based on a lignin-derived spiro diacetal trigger. *J. Mater. Chem. A* **7**, 1233-1243 (2019).

23 Wypych, G. *Handbook of polymers*. (Elsevier, 2022).

24 Duan, C. *et al.* High wear-resistant performance of thermosetting polyimide reinforced by graphitic carbon nitride (g-C3N4) under high temperature. *Compos. Part A-Appl. Sci. Manuf.* **113**, 200-208 (2018).

25 Wang, B. *et al.* High-performance, biobased, degradable polyurethane thermoset and its application in readily recyclable carbon fiber composites. *ACS Sustain. Chem. Eng.* **8**, 11162-11170 (2020).

26 Chieruzzi, M., Miliozzi, A. & Kenny, J. M. Effects of the nanoparticles on the thermal expansion and mechanical properties of unsaturated polyester/clay nanocomposites. *Compos. Part A-Appl. Sci. Manuf.* **45**, 44-48 (2013).
